# TrkAIII signals endoplasmic reticulum stress to the mitochondria in neuroblastoma cells, resulting in glycolytic metabolic adaptation

**DOI:** 10.18632/oncotarget.23618

**Published:** 2017-12-22

**Authors:** Antonietta Rosella Farina, Lucia Cappabianca, Luciana Gneo, Pierdomenico Ruggeri, Andrew Reay Mackay

**Affiliations:** ^1^ Department of Applied Clinical and Biotechnological Sciences, University of L'Aquila, L’Aquila 67100, Italy

**Keywords:** neuroblastoma, TrkAIII, Omi/HtrA2, mitochondria, aerobic glycolysis

## Abstract

Alternative TrkAIII splicing characterises advanced stage metastatic disease and post-therapeutic relapse in neuroblastoma (NB), and in NB models TrkAIII exhibits oncogenic activity. In this study, we report a novel role for TrkAIII in signaling ER stress to the mitochondria in SH-SY5Y NB cells that results in glycolytic metabolic adaptation. The ER stress-inducing agents DTT, A23187 and thapsigargin activated the ER stress-response in control pcDNA SH-SY5Y and TrkAIII expressing SH-SY5Y cells and in TrkAIII SH-SY5Y cells increased TrkAIII targeting to mitochondria and internalisation into inner-mitochondrial membranes. Within inner-mitochondrial membranes, TrkAIII was subjected to Omi/HtrA2-dependent cleavage to tyrosine phosphorylated 45–48kDa carboxyl terminal active fragments, localised predominantly in tyrosine kinase-domain mitochondrial matrix orientation. This stress-induced activation of mitochondrial TrkAIII was associated with increased ROS production, prevented by the ROS scavenger Resveratrol and underpinned by changes in Ca2+ movement, implicating ROS/Ca2+ interplay in overcoming the mitochondrial TrkAIII activation threshold. Stress-induced, cleavage-activation of mitochondrial TrkAIII resulted in mitochondrial PDHK1 tyrosine phosphorylation, leading to glycolytic metabolic adaptation. This novel mitochondrial role for TrkAIII provides a potential self-perpetuating, drug reversible way through which tumour microenvironmental stress may maintain the metastasis promoting “Warburg effect” in TrkAIII expressing NBs.

## INTRODUCTION

Alternative TrkAIII splicing of the neurotrophin receptor gene *TrkA* in neuroblastoma (NB) is characterised by exon 6–7 skipping, associates with advanced stage metastatic disease and post-therapeutic relapse, and in NB models TrkAIII exhibits oncogenic activity and promotes chemotherapeutic resistance [1–8]. The TrkAIII oncoprotein is devoid of the D4 activation-prevention domain [1, 9] and several N-glycosylation sites important for cell surface receptor localisation [1, 10]. As a consequence, TrkAIII is not expressed at the cell surface but accumulates within pre-Golgi membranes and at the centrosome, where it exhibits spontaneous ligand-independent activation. Spontaneous intracellular TrkAIII activation leads to chronic signaling through the IP3K/Akt but not RAS/MAPK pathway and promotes a more stem cell-like, anaplastic, pro-angiogenic, stress-resistant, genetically unstable, tumourigenic and metastatic phenotype [1–3, 6, 7, 11–13]. In NB cell lines, alternative TrkAIII splicing is promoted by a hypoxia mimic, suggesting that it represents a mechanism through which tumour suppressing signals from fully spliced TrkA receptors can switch to tumor promoting signals from TrkAIII within the hypoxic tumour microenvironment [1, 2, 6]. Furthermore, spontaneous activation of TrkAIII within the ERGIC-COP1 compartment and at the centrosome provides novel alternatives to “classical” cell surface oncogenic receptor tyrosine kinase (RTK) signaling and fuels the growing hypothesis that the RTK oncoprotein mislocalization underpins oncogenic activity [11, 14, 15].

Stress within the tumour microenvironment promotes tumour progression by selecting resistant tumour cells that are protected against stress-induced death by conserved physiological stress-protection mechanisms, activated oncogenes and the loss of tumour suppressors. The endoplasmic reticulum stress response (ERSR) represents one such mechanism that is conserved by tumour cells and utilised for adaptation and survival within the stressful tumour microenvironment [16]. The ERSR is activated by the accumulation of damaged, under-glycosylated and/or misfolded proteins within the ER and is induced by hypoxia, acidosis and nutrient deprivation, all of which characterise the tumour microenvironment. Damaged, misfolded and/or aggregated proteins accumulating within the ER competitively bind the ER chaperone Grp78/Bip, which dissociates from the ER stress-response factors ATF6, Ire1α and PERK. These factors are subsequently activated and orchestrate an adaptive response that reduces protein translation, increases ER storage capacity, eliminates damaged proteins, re-folds misfolded proteins, alters metabolism and protects against ER stress-induced death [16, 17].

The ER also communicates with mitochondria via specialised mitochondrial-associated ER membrane (MAM) sites. These sites regulate the flow of Ca^2+^, proteins and lipids between the ER and mitochondria [18, 19]. ER stress causes the release of Ca^2+^ from the ER lumen [20] and increases mitochondrial Ca^2+^ uptake. Mitochondrial Ca^2+^ is critical for respiratory function, optimises respiratory enzyme activity and regulates mitochondrial ROS production [20, 21] but elevated levels of mitochondrial Ca^2+^ have potential to increase mitochondrial ROS production to damaging levels [20–27]. Under such conditions, the fate of mitochondria is regulated by redox enzyme systems, superoxide dismutases, the inter-membrane space serine protease Omi/HtrA2 [28–32] and also by the mitochondrial unfolded protein response (mt-UPR). The mt-UPR activates an independent transcriptional program that enhances mitochondrial survival through metabolic adaptation, proteolytic elimination of damaged proteins and selective elimination of damaged mitochondria [33]. Severe ER stress, however, induces apoptosis by elevating levels of mitochondrial Ca^2+^ and ROS, which either directly open the mitochondrial membrane permeability pore or indirectly promote BAX polymerisation. Under such conditions, mitochondrial survival is also regulated by the expression levels of anti-apoptotic Bcl-2 family proteins and by metabolic adaptation to aerobic glycolysis within the cytosol [21, 28–35].

Malignant tumours, including NB, are characterised by a glycolytic metabolic adaptation termed the “Warburg effect” [36, 37]. This effect, not only provides a selective advantage for tumour cells by increasing glucose uptake to provide carbons for biosynthetic pathways but also promotes micro-environmental stress by increasing the extracellular concentration of lactate, resulting in a reductive acidic microenvironment. Maintenance of this microenvironment further selects stress-resistant tumour cells, is toxic for normal cells and facilitates formation of the cancer stem cell niche required for metastatic progression [38–42].

A greater understanding of the molecular mechanisms through which malignant tumours promote and maintain the “Warburg effect” should provide novel therapeutic ways to reverse its effect and slow tumour progression, as illustrated by metastasis suppressor KISS1 reversal of the Warburg effect [42]. Within this context, we present evidence for a novel stress-induced “Warburg”-promoting role for the TrkAIII oncoprotein in NB cells. We report that TrkAIII signals ER stress to the mitochondria, resulting in glycolytic metabolic adaptation, characterising a novel drug-reversible mechanism through which stress within the tumour microenvironment may maintain the “Warburg effect” in TrkAIII expressing NBs.

## RESULTS

### DTT, A23187 and thapsigargin activate the ERSR in SH-SY5Y cells

**Figure 1:**
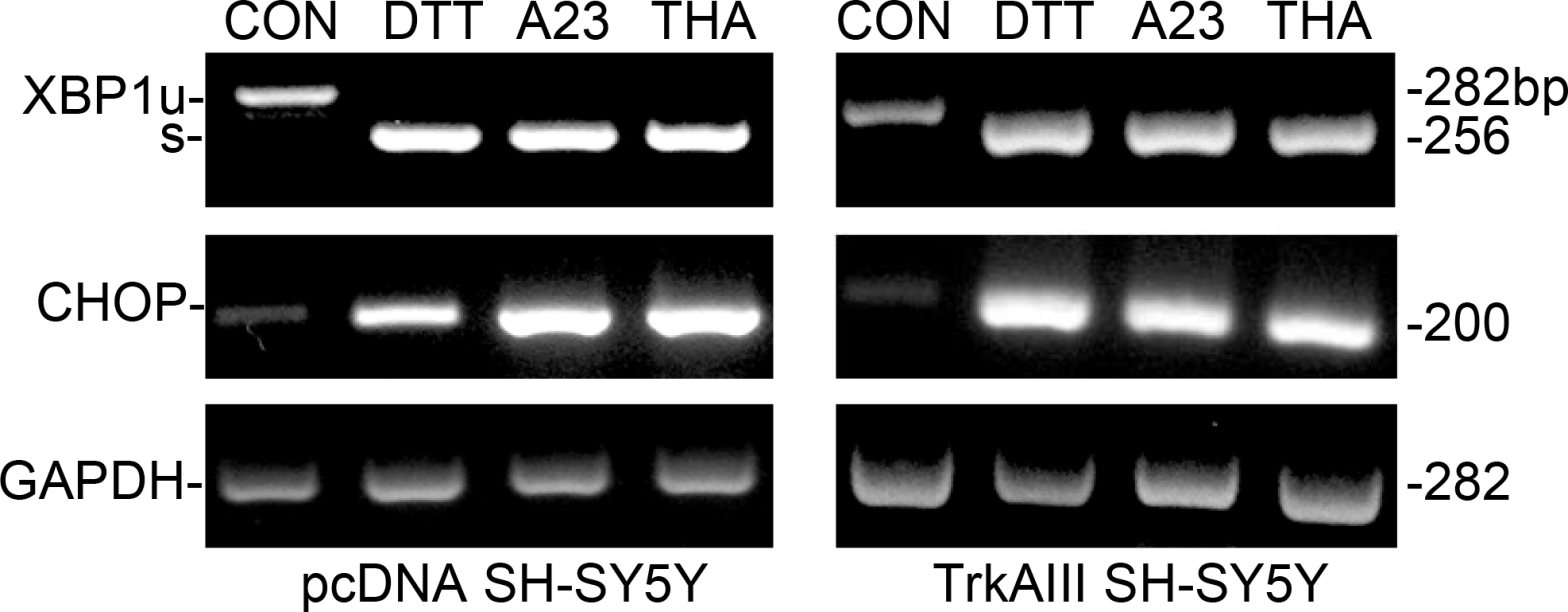
DTT, A23187 and thapsigargin activate the ER stress response RT-PCR demonstrating DTT (5 mM), A23187 (A23, 10 µM) and thapsigargin (THA, 10 ng/ml) induction of unconventional XBP-1s mRNA splicing within 1 hour and stimulation of CHOP mRNA expression within 12 hours, compared to GAPDH mRNA expression, in pcDNA SH-SY5Y and TrkAIII SH-SY5Y cells.

DTT (5 mM), A23187 (10 µM) and thapsigargin (10 ng/ml) all induced unconventional Xbp-1 splicing within 1 hour and increased CHOP mRNA expression within 12 hours in control (pcDNA SH-SY5Y) and TrkAIII SH-SY5Y cells, confirming activation of the ERSR (Figure 1).

### Mitochondrial TrkAIII targeting is enhanced under conditions of ER stress

**Figure 2:**
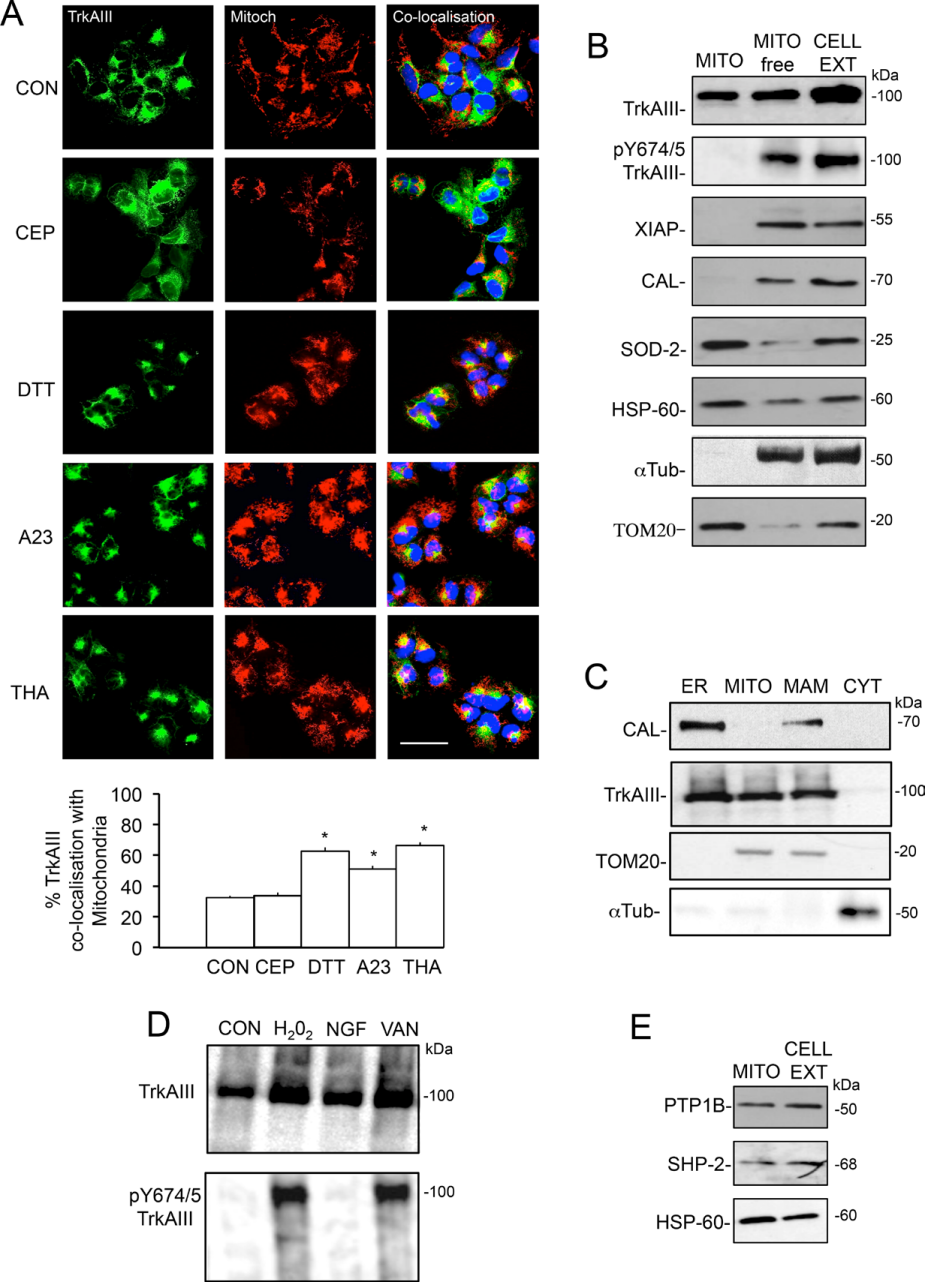
Mitochondrial TrkAIII is inactive under non-stress conditions (**A**) IF micrographs demonstrating TrkAIII expression (green), MitoTracker Red-labelled mitochondria (red) and TrkAIII-mitochondria co-localisation (yellow) in untreated TrkAIII SH-SY5Y cells (CON) and in TrkAIII SH-SY5Y cells treated with CEP-701 (CEP, 100nM), DTT (5 mM), A23187 (A23, 10 µM) and thapsigargin (THA, 10 ng/ml) for 3 hours (bar = 50 µM), plus a histogram demonstrating quantitated differences in TrkAIII co-localisation with mitochondria under the same conditions. Results are displayed as the mean (± s.e.) percentage co-localisation per cell, in 50 cells per group (^*^= statistical significance). (**B**) Western blots demonstrating levels of total TrkAIII (TrkAIII), Y674/675 phosphorylated TrkAIII (pY674/5 TrkAIII), XIAP, calnexin (CAL), SOD-2, HSP-60, α-tubulin (α-Tub) and TOM20 in mitochondria (MITO), mitochondria-depleted cell extracts (MITO-free) and total cell extracts (CELL EXT) from TrkAIII SH-SY5Y cells (20 μg per lane). (**C**) Western blots demonstrating TrkAIII and calnexin (CAL) but not TOM-20 or α-tubulin in purified ER membranes; TrkAIII and TOM20 but not calnexin or α-tubulin in purified mitochondria; TrkAIII, calnexin (CAL) and TOM20 but not α-tubulin (α-Tub) in purified MAMs; and α-tubulin but not TrkAIII, calnexin or TOM20 in membrane-free cytosol (CYT) from TrkAIII SH-SY5Y cells (20 μg/lane). (**D**) Western blots demonstrating the induction of mitochondrial TrkAIII Y674/675 phosphorylation (pY674/5TrkAIII), following 15-minute incubation with H_2_O_2_ and sodium orthovanadate (VAN) but not with NGF (20 μg/lane). (**E**) Western blots demonstrating the presence of PTP1B, SHP-2 and HSP60 in purified mitochondria and whole cell extracts from TrkAIII SH-SY5Y cells (20 μg/lane, right panels).

In IF studies, TrkAIII co-localisation with MitoTracker Red-labelled mitochondria in non-stressed TrkAIII SH-SY5Y cells was significantly enhanced following treatment with DTT (5 mM), A23187 (10 µM) and thapsigargin (10 ng/ml) for 3 hours, from a mean (± s.e.) of 32.6 ± 2.1% in non-stressed TrkAIII SH-SY5Y cells, not significantly reduced by pre-incubation with the TrkA inhibitor CEP-701 (100 nM for 3 hours) [43], to 66.2 ± 2.15% following treatment with DTT, 62.7 ± 2.4% following treatment with A23187 and 51.2 ± 1.7% following treatment with thapsigargin (*p* < 0.0001, df = 98, for all three treatments) (Figure 2A), indicating that ER stress promotes TrkAIII targeting to the mitochondria.

TrkAIII localisation to mitochondria was confirmed by Western blot in density gradient ultracentrifugation purified mitochondria from non-stressed TrkAIII SH-SY5Y cells. Ultracentrifugation purified mitochondria were positive for TrkAIII and the mitochondrial markers TOM-20, Hsp60, PDHK1, cytochrome C and SOD2 but negative for the cytosolic proteins XIAP and α-tubulin and the ER protein calnexin (Figure 2B). TrkAIII was detected in whole cell extracts (CELL EXT) positive for all markers and also in nuclei and mitochondria depleted extracts (MITO-free) positive for XIAP, calnexin and α-tubulin and depleted of SOD-2, Hsp60 and TOM20 (Figure 2B). TrkAIII was constitutively Y490 and Y674/5 phosphorylated in whole cell (CELL EXT) and mitochondrial-depleted (MITO-free) extracts and but was not phosphorylated in mitochondrial extracts (Figure 2B, data shown for TrkAIII Y674/5 phosphorylation only).

In addition to density gradient ultracentrifugation-purified mitochondria, TrkAIII was also detected in purified ER membranes, confirming previous reports [3, 11], and in density gradient ultracentrifugation-purified MAMs but was not detected in membrane-free 100,000 x g ultracentrifugation cytosol fractions (Figure 2C). TrkAIII positive MAMs were positive for TrkAIII, calnexin and TOM20 but not α-tubulin, whereas ER membranes were positive for TrkAIII and calnexin but not TOM20 and α-tubulin, confirming MAM purification as previously reported [44, 45]. TrkAIII positive mitochondria were positive for TOM20 but not calnexin and α-tubulin and membrane-free cytosol was positive for α-tubulin but negative for TrkAIII, TOM20 and calnexin (Figure 2C), indicating that TrkAIII localises to ER membranes, MAMs and mitochondria but not membrane-free cytosol.

Incubation of mitochondria purified from non-stressed TrkAIII SH-SY5Y cells with H_2_O_2_ (500 µM) or sodium orthovanadate (0.1 mM) for 15 minutes, resulted in mitochondrial TrkAIII Y674/5 phosphorylation (Figure 2C, left panel). In contrast, incubation of purified mitochondria with NGF (100 ng/ml for 15 minutes) did not induce mitochondrial TrkAIII tyrosine phosphorylation. In Western blots, the protein tyrosine phosphatases PTP-1B and SHP-2 were detected in mitochondria purified from TrkAIII SH-SY5Y cells and total TrkAIII SH-SY5Y cell extracts (Figure 2E).

### ER stress promotes mitochondrial TrkAIII importation into IMMs and TrkAIII activation

**Figure 3:**
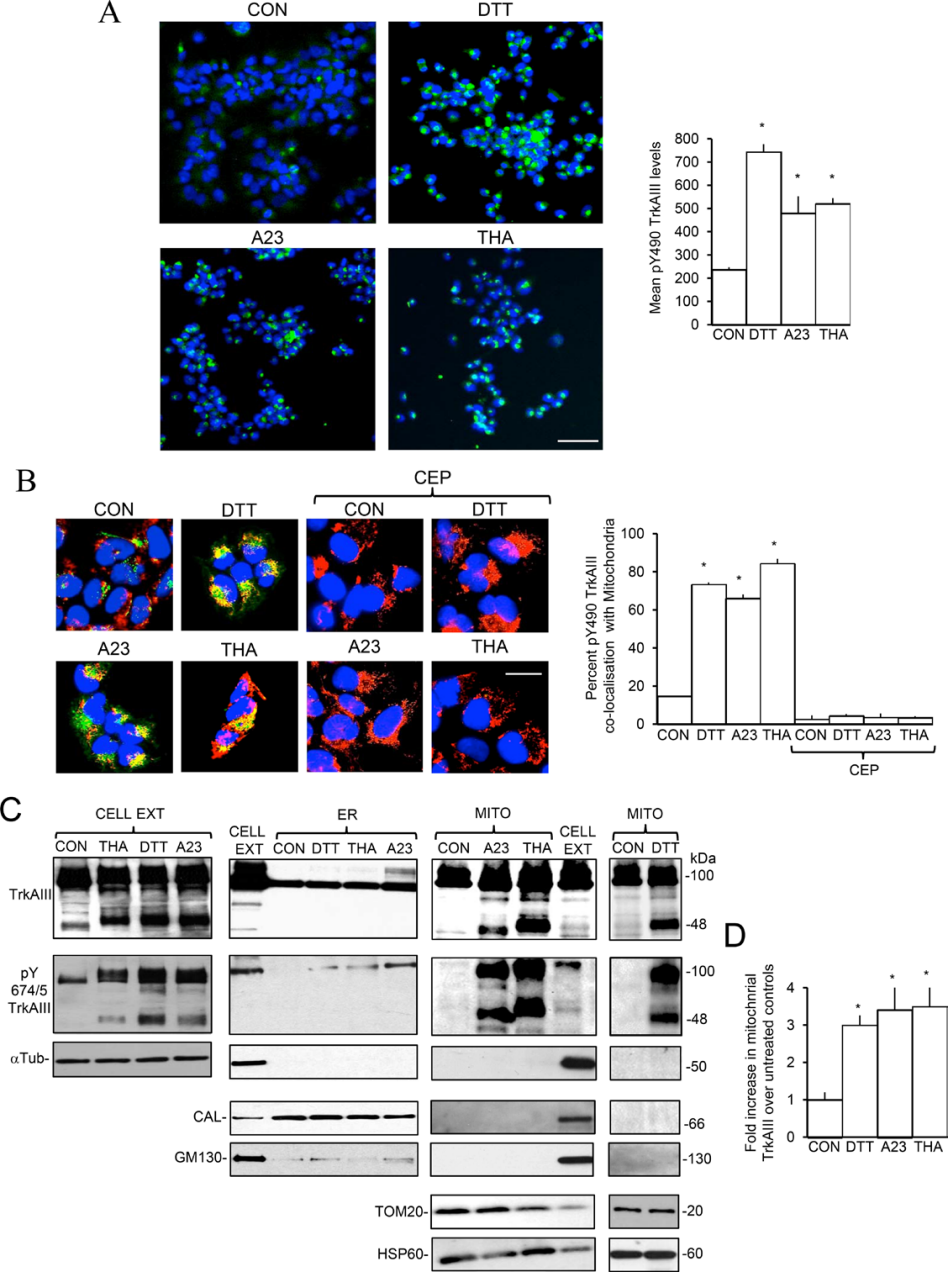
ER stress-induces Mitochondrial Importation and TrkAIII cleavage-activation (**A**) IF micrographs demonstrating increased levels of TrkAIII Y490 phosphorylation (green) in TrkAIII SH-SY5Y cells treated with DTT (5 mM), A23187 (A23, 10 µM) and thapsigargin (THA, 10 ng/ml) for 3 hours compared to untreated control (CON) (bar = 100 μm, nuclei are stained with DAPI) plus a histogram demonstrating quantitative differences in the levels of Y490 phosphorylated TrkAIII (pY490TrkAIII), under these conditions. Results are displayed as the mean (± s.e.) densitometric levels of Y490 phosphorylated TrkAIII per cell, in 50 cells per group. (**B**) IF micrographs demonstrating marked increase in Y490 phosphorylated TrkAIII (green) co-localisation (yellow) with MitoTraker red-stained mitochondria (red) in TrkAIII SH-SY5Y cells treated with DTT (5 mM), A23187 (A23, 10 µM) and thapsigargin (THA, 10 ng/ml) for 3 hours, compared to untreated control (CON) (left 4 panels) and the complete abrogation of TrkAIII Y490 phosphorylation in TrkAIII SH-SY5Y cells pre-incubated with CEP-701 (100 nM) for 3 hours prior to treatment with DTT (5 mM), A23187 (A23, 10 µM) and thapsigargin (THA, 10 ng/ml) for 3 hours, and in untreated controls (CON) (right 4 panels) (bar = 10 μm) plus a histogram demonstrating the differences in percentage Y490 phosphorylated TrkAIII (pY490TrkAIII) co-localisation with mitochondria under these conditions. Results are displayed as the mean (± s.e.) percentage co-localisation per cell, in 20 cells per group (^*^ = statistical significance). (**C**) Western blots demonstrating the effect of TrkAIII SH-SY5Y treatment with DTT (5 mM), A23187 (A23, 10 µM) and thapsigargin (THA, 10 ng/ml) for 6 hours on total TrkAIII and Y674/5 phosphorylated (pY674/5 TrkAIII) in purified α-tubulin positive total cell extracts (CELL EXT), purified calnexin (CAL) positive, GM130 depleted, α-tubulin (αTub) negative ER membranes and purified TOM20 and HSP60 positive, GM130 and calnexin (CAL) negative mitochondria compared untreated controls (CON) (20 μg/lane). (**D**) Histogram demonstrating increased levels of mitochondrial TrkAIII (full length plus cleaved), following treatment with DTT (5 mM), A23187 (A23, 10 µM) and thapsigargin (THA, 10 ng/ml) for 6 hours, compared to untreated controls (CON). Results are displayed as the mean (± s.e.) fold increase over controls, in 4 independent Western blots, normalised with respect to HSP60 (^*^ = statistical significance).

In IF-densitometric studies, treatment of TrkAIII SH-SY5Y cells with DTT (5 mM), A23187 (10 µM) and thapsigargin (10 ng/ml) for 3 hours, significantly increased the levels of Y490 phosphorylated TrkAIII per cell from a mean (± s.e.) of 237.13±20.2 densitometric units per cell in untreated TrkAIII SH-SY5Y cells to 743.9 ± 32.63 in DTT-treated cells, 479 ± 74.14 in A23187-treated cells and 520.8 ± 24.2 in thapsigargin-treated cells (*p* < 0.0001, df = 98, for all three treatments) (Figure 3A). All three treatments also significantly increased Y490 phosphorylated TrkAIII (pY490 TrkAIII) co-localisation with MitoTracker-red labelled mitochondria from a mean (±s.e.) percent per cell of 14.8 ± 2.4% in non-stressed TrkAIII SH-SY5Y cells to 73.44 ± 2.84% following DTT-treatment, 66 ± 1.94% following A23187-treatment and 84.3 ± 2.24% following thapsigargin-treatment (*p* < 0.0001, df = 38, for all three treatments) (Figure 3B). Pre-incubation with CEP-701 (100 nM for 3 hours) completely abrogated TrkAIII Y490 phosphorylation in untreated, DTT, A23187 and thapsigargin-treated TrkAIII SH-SY5Y cells (Figure 3B, right 4 panels).

**Figure 4:**
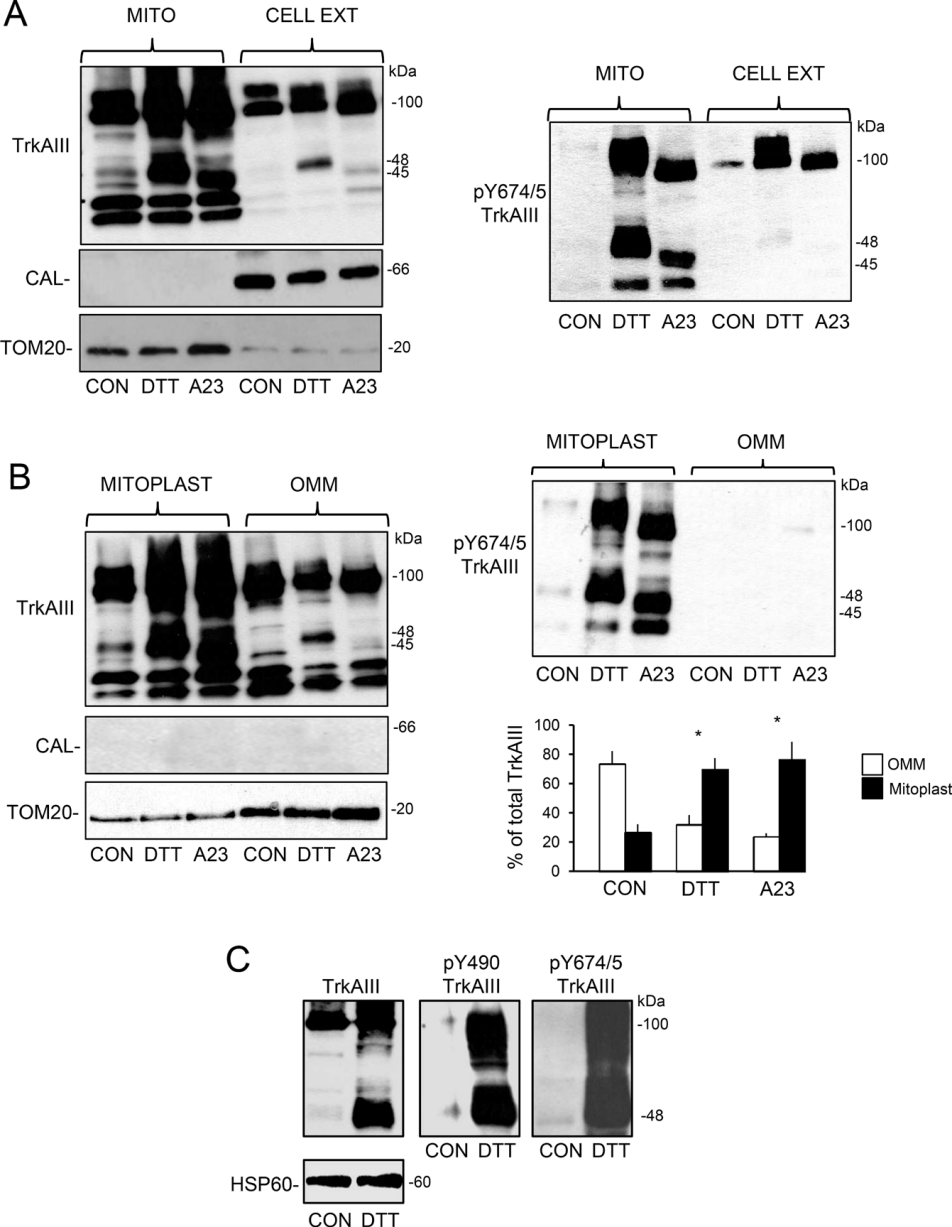
ER stress promotes mitochondrial internalisation and cleavage-activation (**A**) Western blots demonstrating enrichment of un-cleaved and cleaved TrkAIII (TrkAIII), enrichment of Y674/5 phosphorylated TrkAIII (pY674/5 TrkAIII) and enrichment of TOM20 in calnexin (CAL) negative mitochondria (MITO), compared to calnexin (CAL) and reduced TOM20 positive whole cell extracts (CELL EXT) from TrkAIII SH-SY5Y cells treated for 6 hours with DTT (5 mM) and A23187 (A23, 10 µM). (**B**) Western blots demonstrating enrichment of un-cleaved and cleaved TrkAIII (TrkAIII), enrichment of Y674/5 phosphorylated TrkAIII (pY674/5 TrkAIII), combined with reduced TOM20 levels, in calnexin negative mitoplasts compared to TOM20-enriched OMMs from TrkAIII SH-SY5Y cells treated for 6 hours with DTT (5 mM) and A23187 (A23, 10 µM), plus a histogram quantifying the re-distribution of TrkAIII (full length plus cleaved) from OMMs (white) to mitoplasts (black), following 6 hour treatment with DTT (5 mM) and A23187 (A23, 10 µM). Results are displayed as the mean (± s.e.) percent of total TrkAIII, from the densitometric analysis of 4 independent Western blots (^*^ = statistical significance). (**C**) Western blots demonstrating DTT (5 mM for 6 hours) induction of TrkAIII cleavage, TrkAIII Y674/5 phosphorylation and TrkAIII Y490 phosphorylation in mitochondria from TrkAIII SH-SY5Y cells.

Western blot comparisons of total cell extracts (CELL EXT), purified ER membranes and ultracentrifugation-purified mitochondria from untreated, DTT, A23187 and thapsigargin-treated TrkAIII SH-SY5Y cells, revealed that all three treatments induced TrkAIII Y490 and Y674/5 phosphorylation, associated with TrkAIII cleavage to a major 48kDa CT Y490 and Y674/5 phosphorylated fragment for DTT and thapsigargin and a major 45kDa Y490 and Y674/5 phosphorylated CT fragment for A23187 (Figure 3C). TrkAIII cleavage-activation detected in whole cell extracts was not detected in purified ER membranes and was enriched in purified mitochondria (Figure 3C, data not displayed for Y490 TrkAIII phosphorylation). In mitochondria, A23187-treatment also reduced the molecular size of mitochondrial TrkAIII to 95kDa (Figure 3C and Figure 4). Densitometric analysis, confirmed that DTT had significantly increased mitochondrial TrkAIII levels (uncleaved plus cleaved) by 3 ± 0.6 fold (*p* = 0.016, df = 6), A23187 by 3.4 ± 0.8 fold (*p* = 0.031, df = 6) and thapsigargin by 3.5 ± 0.9 fold (*p* = 0.039, df = 6) (Figure 3D), confirming IF observations (Figure 2A). Similar results were observed in 3 independent TrkAIII SH-SY5Y cell lines (data not shown).

Focussing on the effect of DTT and A23187, Western blot comparisons of whole cell extracts and purified mitochondrial clearly demonstrated enrichment of both un-cleaved and cleaved Y674/5 phosphorylated TrkAIII in mitochondria (Figure 4A). Furthermore, a densitometric comparison of cleaved and un-cleaved TrkAIII levels in purified mitoplasts and outer mitochondrial membranes (OMMs) in 4 independent Western blots, revealed that DTT had significantly reduced OMM levels of 100kDa TrkAIII from a mean (± s.e.) of 73.5.6 ± 8.7% to 32 ± 6.5% (*P* = 0.0087, df = 6) and significantly increased mitoplast levels of TrkAIII from 26.5 ± 5.6% to 69.8 ± 7.6% (*p* = 0.0034, df = 6) (Figure 4B), confirming TrkAIII translocation from the OMM to the IMM. Although not shown, TrkAIII Y674/5 phosphorylation was paralleled by Y490 phosphorylation under all conditions. This is illustrated in Figure 4C that demonstrates DTT-induced TrkAIII Y674/5 and Y490 phosphorylation in mitochondria purified from TrkAIII SH-SY5Y cells. TrkAIII Y490 rather that Y674/5 phosphorylation was assessed by IF, as the pY674/5 TrkA antibody was not suitable for IF. It also should be noted that the pY674/5 TrkA and pY490 TrkA antibodies might also recognise phosphorylated TrkB but neither recognised similar proteins in non-transfected or pcDNA SH-SY5Y cells in either IF or Western blots [1].

### Topographical analysis of mitochondrial TrkAIII under non-stress and ER stress conditions

**Figure 5:**
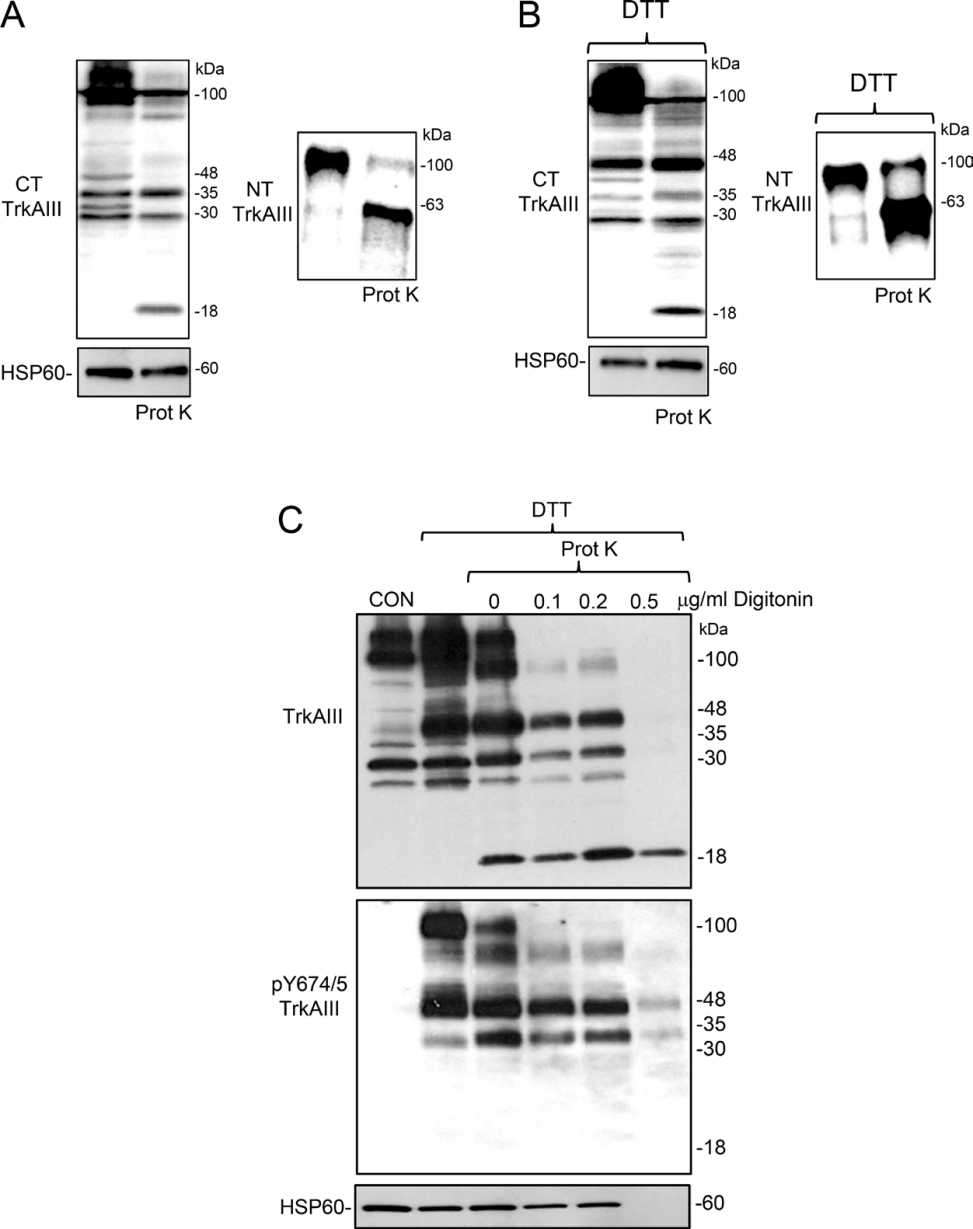
DTT-induces mitochondrial TrkAIII cleavage to an active IMM-associated 48kDa CT fragmented in Mitochondrial matrix orientation (**A**) Western blots demonstrating proteinase K (Prot K) digestion of TrkAIII in mitochondria from non-stressed TrkAIII SH-SY5Y cells to predominant C-terminal 18kDa (CT TrkAIII) and N-terminal 63kDa (NT TrkAIII) fragments, compared to TrkAIII in non-digested mitochondria (20 μg/lane). (**B**) Western blots demonstrating proteinase K (Prot K) cleavage of full length TrkAIII to 18kDa CT (CT TrkAIII) and 63kDa NT fragments (NT TrkAIII) but not DTT-induced 48kDa or constitutive 30-35kDa CT TrkAIII cleavage fragments in mitochondrial purified from DTT-treated (5mM for 6 hours) TrkAIII SH-SY5Y cells. (**C**) Western blots demonstrating proteinase K (Prot K) digestion of full length TrkAIII (TrkAIII) and full-length tyrosine phosphorylated TrkAIII (pY674/5 TrkAIII) but not DTT-induced 48kDa or constitutive 30-35kDa CT TrkAIII cleavage fragments in mitochondria from DTT-treated TrkAIII SH-SY5Y cells in the absence of digitonin (0), increased proteinase K digestion of full length TrkAIII but not DTT-induced 48kDa, constitutive 30-35kDa and proteinase K generated 18kDa CT TrkAIII cleavage fragments and HSP60, in the presence of 0.1-0.2 μg/ml digitonin and complete proteinase K digestion of DTT-induced 48kDa, constitutive 30-35kDa CT TrkAIII cleavage fragments and HSP60 but not the 18kDa OMM-associated TrkAIII CT fragment, in the presence of 0.5 μg/ml digitonin.

The topography of mitochondrial TrkAIII was assessed by proteinase K digestion. Proteinase K digestion of mitochondria purified from non-stressed TrkAIII SH-SY5Y cells, resulted in degradation of the majority of TrkAIII to 18kDa carboxyl terminal (CT) and 63kDa amino terminal (NT) fragments both of which remained associated with mitochondria, suggesting a predominant OMM localisation for TrkAIII under non-stress conditions (Figure 5A). Mitochondria from non-stressed cells also contained 30–35 kDa CT TrkAIII fragments (Figure 5A) that were not digested by proteinase K alone and were only degraded by proteinase K in the presence of IMM permeabilizing digitonin concentrations, suggesting a mitochondrial matrix localisation (Figure 5C).

In the absence of digitonin, proteinase K also degraded 100kDa TrkAIII to 18kDa CT and 63kDa NT fragments in mitochondria purified from DTT-treated TrkAIII SH-SY5Y cells but did not degrade the DTT-induced 48kDa CT Y674/5 phosphorylated TrkAIII cleavage-fragment or the constitutive 30-35kDa CT TrkAIII fragments (Figure 5B and 5C). At OMM permeabilizing digitonin concentrations, proteinase K did not completely degrade 100kDa TrkAIII (Figure 5B and 5C), suggesting increased internalisation of un-cleaved TrkAIII into IMM structures protected from degradation (i.e. cristae) and also failed to degrade either the DTT-induced 48kDa or constitutive 30-35kDa TrkAIII cleavage-fragments. These fragments were only degraded in the presence of IMM permeabilizing digitonin concentrations (0.5 μg/ml), suggesting predominant localisation within the mitochondrial matrix (Figure 5C). Interestingly, digitonin did not promote further degradation of the 18kDa TrkAIII fragment generated by proteinase K. This suggests that this fragment, which contains proteinase K degradation sites, may be protected by insertion within the OMM bilayer.

### Omi/HtrA2 is involved in stress-induced mitochondrial TrkAIII importation and cleavage-activation

**Figure 6:**
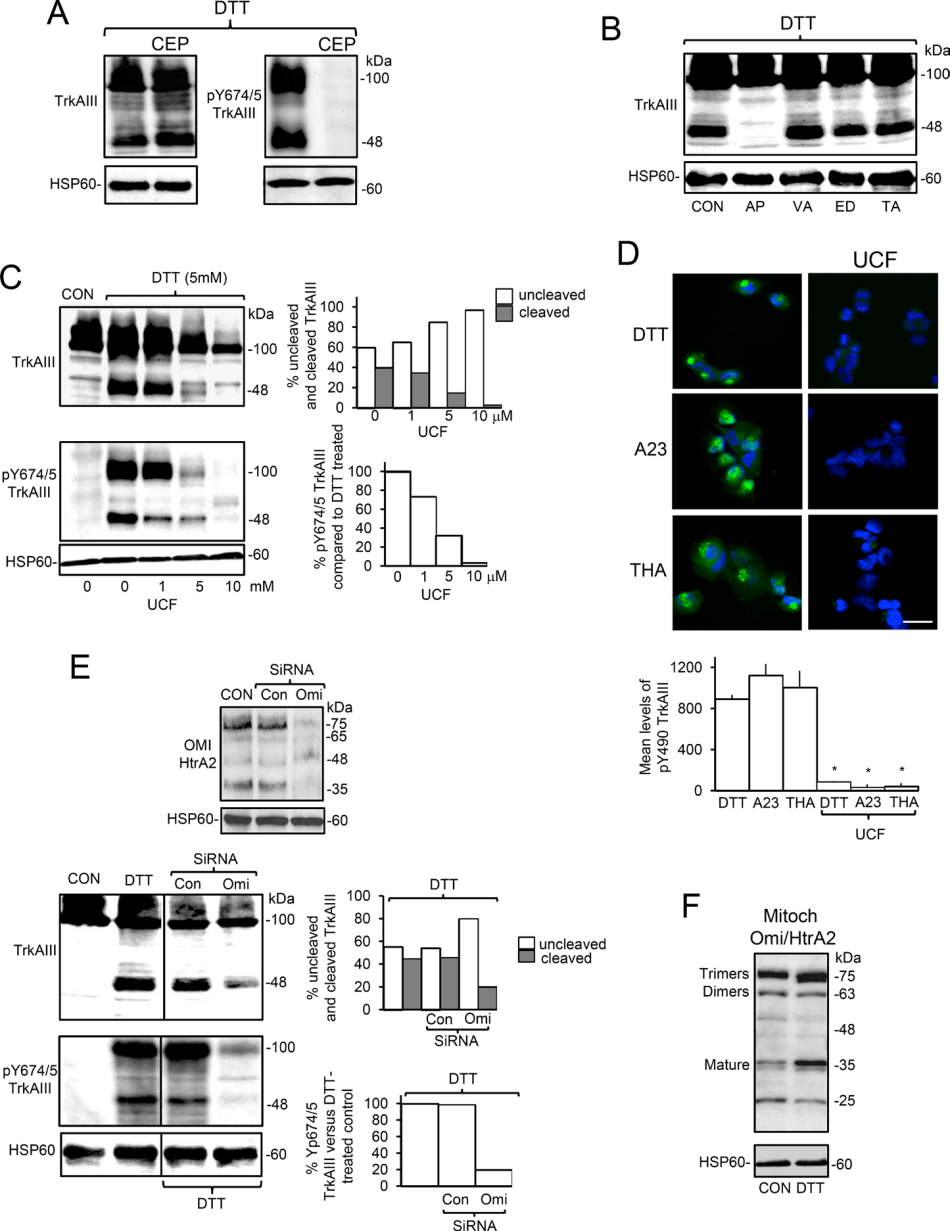
ER stress induced activation of mitochondrial TrkAIII is Omi/HtrA2-mediated and cleavage-dependent (**A**) Western blot demonstrating CEP-701 (CEP, 100 nM, 3 hour pre-incubation) inhibition of DTT-induced (5 mM for 6 hours) TrkAIII Y674/675 phosphorylation (pTrkAIII) but not cleavage (TrkAIII) in mitochondria from TrkAIII SH-SY5Y cells (20 μg/lane). (**B**) Western blot demonstrating the inhibition of DTT-induced (5mM for 6 hours) TrkAIII cleavage (TrkAIII) by aprotinin (10 µM, AP) but not by z-VAD-fmk (10 µM, VA) EDTA (1mM, ED) or TAPI-2 (100µM, TA) in mitochondria from TrkAIII SH-SY5Y cells (20 μg/lane). (**C**) Western blots showing dose-dependent Ucf-101 (UCF, 0-10 µM) inhibition of DTT-induced (5 mM for 6 hours) cleavage (TrkAIII) and Y674/675 phosphorylation (pY674/5 TrkAIII) of TrkAIII in mitochondria from TrkAIII SH-SY5Y cells. HSP60 levels are displayed as loading controls (20 μg/lane), plus a histogram demonstrating densitometric quantification of the adjacent Western blots. (**D**) IF micrographs demonstrating Ucf-101 (UCF 10 mm) inhibition of TrkAIII Y490 phosphorylation induced by DTT (5 mM), A23187 (A23, 10 µM) and thapsigargin (THA, 10 ng/ml) (3 hour-treatment) (bar = 50 µM) plus a histogram demonstrating densitometric quantification of Ucf-101 inhibition of DTT-induced Y490TrkAIII phosphorylation. Results are displayed as the mean (± s.e.) level of Y490 phosphorylated TrkAIII per cell, 20 cells per group (^*^ = statistical significance). (**E**) Western blots demonstrating knockdown of Omi/HtrA2 by Omi/HtrA2-specific (Omi siRNA) but not control siRNAs (Con SiRNA) (upper panels), relative to HSP60 in mitochondria from TrkAIII SH-SY5Y cells (20 μg/lane). Western blots demonstrating reduction in both DTT-induced mitochondrial TrkAIII cleavage (TrkAIII) (middle panels) and Y674/5 phosphorylation (pY674/5 TrkAIII) (bottom panels) compared to HSP60 levels, following siRNA Omi/HtrA2 knockdown but not in control siRNA-treated cells (Cont siRNA) (20 μg/lane) plus histograms demonstrating densitometric quantification of the reduction in DTT-induced TrkAIII cleavage and Y674/5 tyrosine phosphorylation following Omi siRNA knockdown. Results are displayed as the percentage change in un-cleaved and cleaved TrkAIII and percentage change in Y674/5 phosphorylation with respect to controls. (**F**) Western blot demonstrating increased levels of polymerised and mature OMI/HtrA2 in mitochondria purified from DTT-treated (5 mM for 6 hours) compared to untreated (CON) TrkAIII SH-SY5Y cells (20 μg/lane).

Focussing on the effects of DTT, pre-incubation of TrkAIII SH-SY5Y cells with CEP-701 (100 nM, for 3 hours) prevented DTT-induced tyrosine phosphorylation but not cleavage of mitochondrial TrkAIII, confirming that stress-induced cleavage of mitochondrial TrkAIII does not depend upon TrkAIII activity (Figure 6A). DTT-induced cleavage of mitochondrial TrkAIII was prevented by aprotinin (10 μg/ml, AP) (Figure 6B) and also by the Omi/HtrA2 serine protease inhibitor Ucf-101 [46] (Figure 6C) but was not prevented by the caspase inhibitor z-VAD-fmk (10 µM), the MMP inhibitor EDTA (10 mM) or the ADAMs inhibitor TAPI-2 (20µM) [47] (Figure 6B). Ucf-101 inhibited DTT-induced TrkAIII cleavage in a dose-dependent manner from 40% to 3% and inhibited mitochondrial TrkAIII phosphorylation also in a dose-dependent manner by up to 96.5% (Figure 6C). In IF studies, TrkAIII SH-SY5Y pre-incubation with Ucf-101 (10µM, for 3 hours) also significantly reduced DTT-induced TrkAIII Y490 phosphorylation from a mean (± s.e.) densitometric units/cell of 891.3 ± 40.2 to 87.3 ± 5, reduced A23187-induced TrkAIII Y490 phosphorylation from 1123 ± 111.3 to 35.4 ± 25 and reduced thapsigargin-induced TrkAIII Y490 phosphorylation from 1005.2 ± 161 to 43 ± 30 (*p* < 0.0001, n=38, for all three treatments) (Figure 6D). SiRNA knockdown of mitochondrial Omi/HtrA2 (50 nM for 48 hours) (Figure 6E) reduced DTT-induced cleavage of mitochondrial TrkAIII from 45% to 20% and reduced DTT-induced TrkAIII Y674/5 phosphorylation by 80% (Figure 6E). Ucf-101 also reduced levels of mitochondrial TrkAIII relative to HSP60, in DTT-treated TrkAIII SH-SY5Y cells, suggesting a role for Omi/HtrA2 in stress-induced TrkAIII mitochondrial importation (Figure 6C). DTT increased the mitochondrial levels of polymerised and mature Omi/HtrA2 in TrkAIII SH-SY5Y cells (Figure 6F). Together these data implicate Omi/HtrA2 in ER stress-induced TrkAIII mitochondrial importation and cleavage-activation.

### Stress-induced mitochondrial TrkAIII activation is ROS-dependent

**Figure 7:**
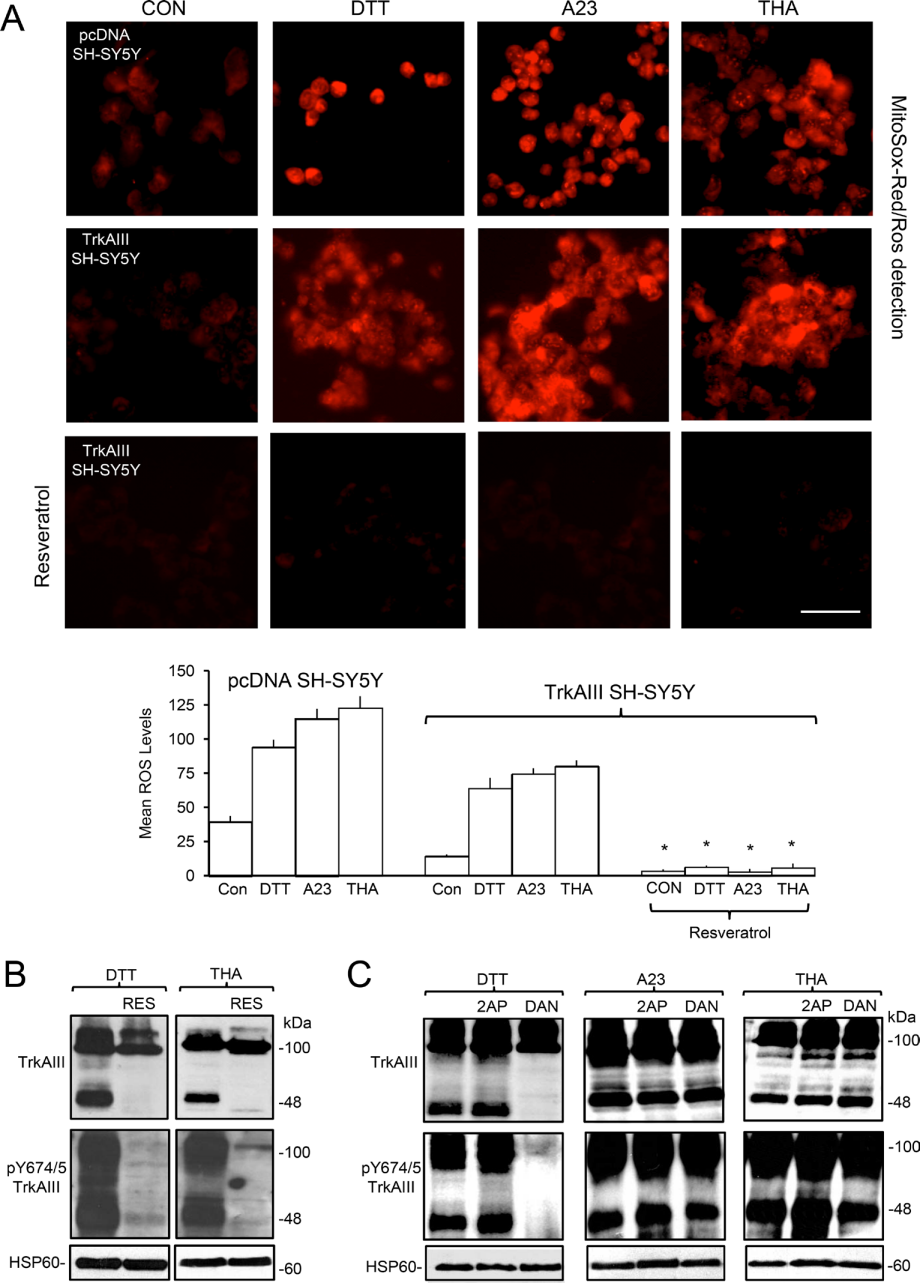
ER stress-induced activation of mitochondrial TrkAIII is ROS-dependent (**A**) Micrographs, under identical exposure conditions, demonstrating increased ROS production (MitoSox-Red/ROS detection) in pcDNA SH-SY5Y and TrkAIII SH-SY5Y cells following 1 hour treatment with DTT (5 mM), A23187 (A23, 10 µM) and thapsigargin (THA, 10 ng/ml) and the abrogation of this effect in TrkAIII SH-SY5Y cells pre-incubated with Resveratrol (100 µM) (bar = 100 μm) plus a histogram demonstrating densitometric quantification of DTT, A23187 (A23) and thapsigargin (THA) stimulation of ROS in pcDNA and TrkAIII SH-SY5Y cells and Resveratrol prevention of this effect in TrkAIII SH-SY5Y. Results are displayed as mean (± s.e.) densitometric units per cell, 50 cells per group (^*^ = statistical significance). (**B**) Western blots demonstrating Resveratrol (100 µM, RES) inhibition of TrkAIII cleavage (TrkAIII) and Y674/5 phosphorylation (pY674/5 TrkAIII) induced by DTT (5 mM for 6 hours) and thapsigargin (THA, 10ng/ml for 6 hours) in mitochondria purified from TrkAIII SH-SY5Y cells (20 μg/lane). (**C**) Western blots demonstrating the inhibitory effect of 3 hour pre-incubation of TrkAIII SH-SY5Y cells with dantrolene (10 µM, DAN) on TrkAIII cleavage (TrkAIII) and Y674/675 phosphorylation (pY674/5 TrkAIII) induced by DTT (5 mM for 6 hours) but not by A23187 (A23, 10 µM for 6 hours) or thapsigargin (THA, 10ng/ml for 6 hours), in mitochondria from TrkAIII SH-SY5Y cells, plus no inhibition of DTT (5mM for 6 hours), A23187 (A23, 10 µM for 6 hours) and thapsigargin (THA, 10ng/ml for 6 hours)-induced TrkAIII cleavage (TrkAIII) and Y674/675 phosphorylation (pY674/5 TrkAIII) in mitochondria from TrkAIII SH-SY5Y cells pre-incubated with 2-APB (2AP, 100 µM for 3 hours) (20 μg/lane).

In fluorescence MitoSox-Red assays, DTT (5 mM), A23187 (10 µM) and thapsigargin (10 ng/ml) all significantly increased ROS levels within 1-hour in pcDNA SH-SY5Y from a mean (± s.e.) densitometric units/cell of 39.3 ± 4.4 to 94.1 ± 5.4 following DTT-treatment, 115±7.2 following A23187-treatment and 123 ± 8.4 following thapsigargin-treatment (*p* < 0.0001, df = 98, for all three treatments) and in TrkAIII SH-SY5Y cells from 14.3 ± 1.2 to 63.9 ± 7.8 following DTT-treatment, 74.4 ± 4.2 following A23187-treatment and 80 ± 4.4 following thapsigargin-treatment (*p* < 0.0001, df = 98, for all three treatments) (Figure 7A). Pre-incubation with the ROS scavenger Resveratrol [48] (100 µM for 12 hours) significantly reduced ROS levels in DTT, A23187 and thapsigargin-treated TrkAIII SH-SY5Y cells by > 90% (*p* < 0.0001 for all treatments) (Figure 7A). In Western blots, pre-incubation with Resveratrol completely abrogated DTT and thapsigargin-induced mitochondrial TrkAIII cleavage and tyrosine phosphorylation (Figure 7B, A23187 not tested). These data add to H_2_O_2_ activation of mitochondrial TrkAIII (Figure 2D), suggesting a central role for ROS in stress-induced mitochondrial TrkAIII cleavage- activation.

### Ca2^*+*^ underpins stress-induced mitochondrial TrkAIII activation

Stress-activation of mitochondrial TrkAIII was induced by a Ca^2+^ ionophore (A23187) [49], a SERCA Ca^2+^ pump inhibitor (thapsigargin) [50] and by a ryanodine receptor Ca^2+^ channel agonist (DTT) [51], implicating Ca^2+^. Ryanodine receptor Ca^2+^ channel involvement in DTT-induced mitochondrial TrkAIII cleavage-activation was confirmed using the ryanodine receptor inhibitor dantrolene [52]. Pre-incubation with dantrolene (10 µM, for 3 hours) abrogated DTT-induced mitochondrial TrkAIII cleavage and Y674/5 phosphorylation (Figure 7C). However, we were unable to successfully knock down ryanodine receptor expression in TrkAIII SH-SY5Y cells to further confirm this. In contrast, dantrolene did not inhibit mitochondrial TrkAIII cleavage-activation induced by either A23187 or thapsigargin, consistent with the ryanodine receptor-independent actions of these agents [49, 50]. Pre-incubation with the IP3 receptor Ca^2+^ channel inhibitor 2-APB (100 µM, for 3 hours) [53] did not inhibit DTT, A23187 or thapsigargin-induced mitochondrial TrkAIII cleavage-activation (Figure 7C). These data implicate the ryanodine receptor in DTT-induced but not in A23187 or thapsigargin-induced mitochondrial TrkAIII cleavage-activation.

### Stress-induced mitochondrial TrkAIII activation results in PDHK1 tyrosine phosphorylation

**Figure 8:**
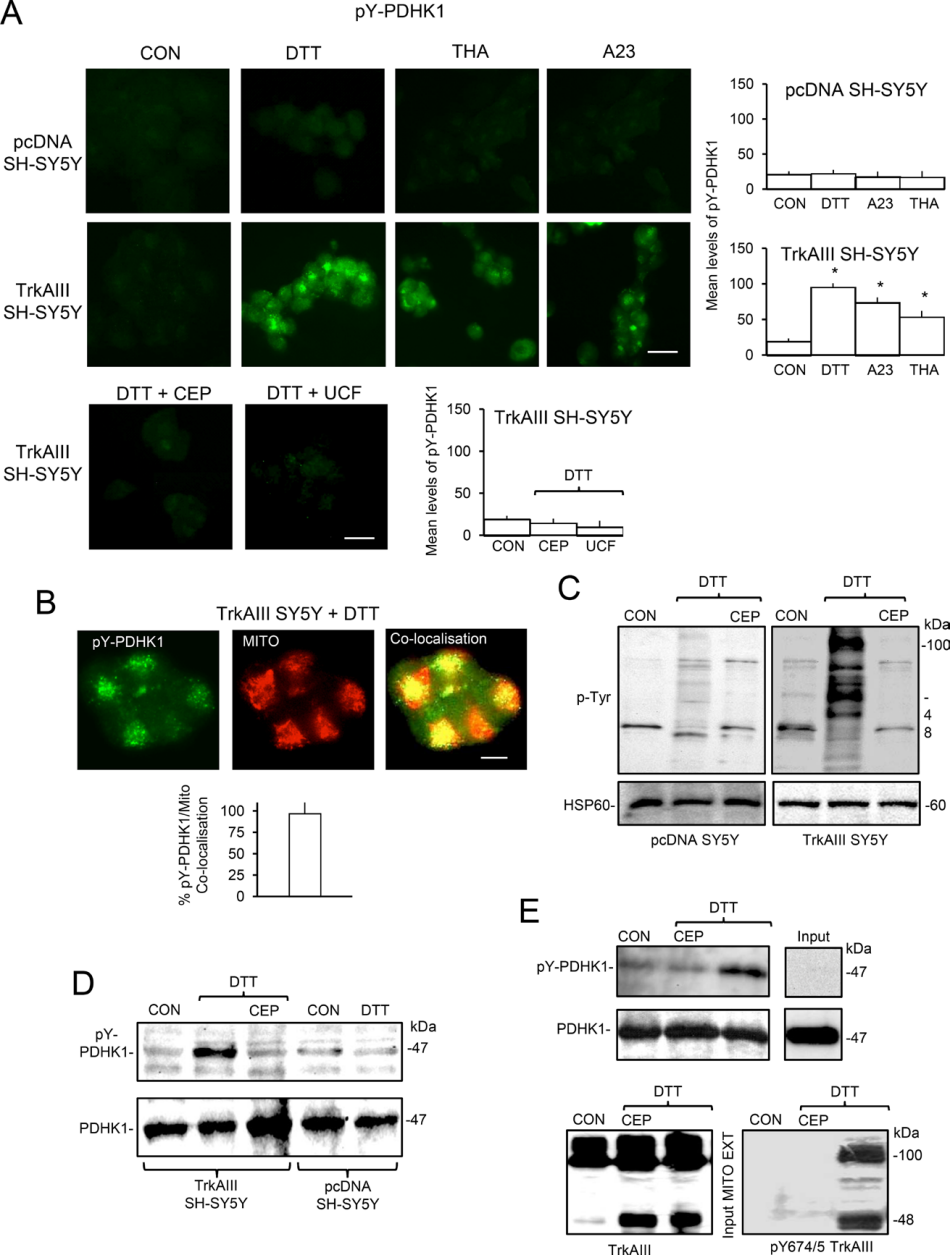
Mitochondrial TrkAIII phosphorylates PDHK1 (**A**) IF micrographs demonstrating the induction of PDHK1 tyrosine phosphorylation (pY-PDHK1, green) in TrkAIII SH-SY5Y cells (lower 4 panels) but not pcDNA SH-SY5Y cells (upper 4 panels) treated for 3 hours with DTT (5mM) A23187 (A23, 10 µM) and thapsigargin (THA, 10 ng/ml), inhibition of DTT-induced PDHK1 tyrosine phosphorylation (green) in TrkAIII SH-SY5Y cells pre-incubated with CEP-701 (100 nM for 3 hours, DTT + CEP) and Ucf-101 (10 µM for 3 hours DTT + UCF) (bottom 2 panels) (bar = 50 μm), plus histograms demonstrating densitometric quantification of PDHK1 phosphorylation levels in TrkAIII SH-SY5Y cells treated with DTT, A23187 and thapsigargin compared to pcDNA SH-SY5Y cells and TrkAIII SH-SY5Y cells pre-incubated with CEP-701 and Ucf-101. Results are displayed as the mean (± s.e.) densitometric units per cell, 20 cells per group (^*^ = statistical significance). (**B**) IF micrographs demonstrating tyrosine phosphorylated PDHK1 (pY-PDHKI, green), MitoTracker-Red labelled mitochondria (red) and pY-PDHK1 co-localisation with mitochondria (yellow), in TrkAIII SH-SY5Y cells treated for 3 hours with DTT (5 mM) (bar=10 µM) plus a histogram displaying quantification of the level of pY-PDHK1 co-localisation with mitochondria. Results are displayed as the mean (± s.e.) percentage co-localisation per cell, in 20 cells. (**C**) Western blots demonstrating increased levels of tyrosine phosphorylated proteins in mitochondria purified from DTT-treated (5 mM, 6 hours) TrkAIII SH-SY5Y cells compared to untreated TrkAIII SH-SY5Y cells, DTT-treated TrkAIII SH-SY5Y cells pre-incubated with CEP-701 (CEP, 100nM for 3 hours), untreated and DTT-treated (5 mM) pcDNA SH-SY5Y cells pre-incubated with or without CEP-701 (20 μg/lane). (**D**) Western blots demonstrating induction of PDHK1 tyrosine phosphorylation (pY-PDHK1) in mitochondria purified from DTT-treated (5mM for 6 hours) TrkAIII SH-SY5Y cells, compared to untreated and DTT-treated pcDNA SH-SY5Y cells or DTT-treated TrkAIII SH-SY5Y cells pre-incubated with CEP-701 (CEP, 100nM for 3 hours) (20 μg/lane). (**E**) An *in vitro* tyrosine kinase assay, demonstrating increased tyrosine phosphorylation of recombinant PDHK1 by TrkAIII immunoprecipitated from DTT-treated TrkAIII SH-SY5Y mitochondria compared to TrkAIII immunoprecipitated from untreated TrkAIII SH-SY5Y mitochondria and CEP-701 (CEP, 100 nM) inhibition of PDHK1 tyrosine phosphorylation by TrkAIII immunoprecipitated from DTT-treated TrkAIII SH-SY5Y mitochondria. Input levels of recombinant PDHK1, which was not detected by the anti-Y-phos-PDHK1 antibody, are presented in the right hand panels and input levels of total (TrkAIII) and tyrosine phosphorylated TrkAIII (pY674/5 TrkAIII) in mitochondrial extracts from control and DTT-treated TrkAIII SH-SY5Y cells in the presence or absence of CEP-701 (CEP) are displayed below (input MITO EXT).

In IF-densitometric studies, TrkAIII SH-SY5Y treatment with DTT (5 mM), A23187 (10 µM) and thapsigargin (10 ng/ml) for 3 hours, significantly increased the levels tyrosine phosphorylated PDHK-1 from a mean (± s.e.) densitometric units/cell of 18.9 ± 4.4 in untreated TrkAIII SH-SY5Y cells to 94.44 ± 5.4 following DTT-treatment, 73.8 ± 7.23 following A23187-treatment and 53.7±8.4 following thapsigargin-treatment (*P* < 0.0001, df = 38, for all three treatments) (Figure 8A). In contrast, DTT, A23187 and thapsigargin did not induce PDHK-1 tyrosine phosphorylation in pcDNA SH-SY5Y cells (Figure 8A, top panels and histograms). Focussing on DTT-treatment, pre-incubation of TrkAIII SH-SY5Y cells with CEP-701 (100 nM, for 3 hours) or Ucf-101 (10 µM, for 3 hours) completely abrogated DTT-induced PDHK1 tyrosine phosphorylation (Figure 8A, bottom panels and histogram). Co-localisation of phosphorylated PDHK-1 with MitoTraker-Red-labelled mitochondria was quantified as 95% in DTT-treated TrkAIII SH-SY5Y cells (Figure 8B). Under conditions of ER stress, phosphorylated PDHK1 exhibited a centralised clustered distribution (Figure 8A and 8B), similar to Y490 phosphorylated TrkAIII.

In Western blots, mitochondria purified from DTT-treated TrkAIII SH-SY5Y cells exhibited marked increase in mitochondrial protein tyrosine phosphorylation that was not detected in mitochondria from cells pre-incubated with CEP-701 (100 nm for 3 hours) or in mitochondria from pcDNA SH-SY5Y cells (Figure 8C). Western blots also detected tyrosine phosphorylated PDHK1 in mitochondria purified from DTT-treated TrkAIII SH-SY5Y cells but not in mitochondria from DTT-treated pcDNA SH-SY5Y cells or from DTT-treated TrkAIII SH-SY5Y and pcDNA SH-SY5Y cells pre-incubated with CEP-701 (100 nM for 3 hours) (Figure 8D). These data implicate TrkAIII activity in the DTT-induced tyrosine phosphorylation of mitochondrial proteins and PDHK1.

In an *in vitro* tyrosine kinase assay, tyrosine phosphorylated TrkAIII immunoprecipitated from mitochondria from DTT-treated TrkAIII SH-SY5Y cells, tyrosine phosphorylated exogenous recombinant PDHK1 *in vitro* to a greater degree in the absence of CEP-701 compared to the presence of CEP-701 (100 nM) and also compared to TrkAIII immunoprecipitated from mitochondria from untreated TrkAIII SH-SY5Y cells (Figure 8E). This suggests that PDHK1 is a novel TrkAIII substrate.

### DTT, A23187 and thapsigargin promote glycolysis in TrkAIII SH-SY5Y cells

In glycolysis assays, lactate production integrated with growth over 24 hours did not significantly differ between non-stressed pcDNA SH-SY5Y and TrkAIII SH-SY5Y cells (*P* = 0.357 df = 10). DTT, A23187 and thapsigargin-treatment promoted extensive death of pcDNASH-SY5Y but not TrkAIII SH-SY5Y cells but did prevent TrkAIII SH-SY5Y growth over the 24-hour assay period, whereas non-stressed TrkAIII SH-SY5Y cells exhibited a growth rate of 3.8 ± 0.5% and more than doubled within 24 hours.

**Figure 9:**
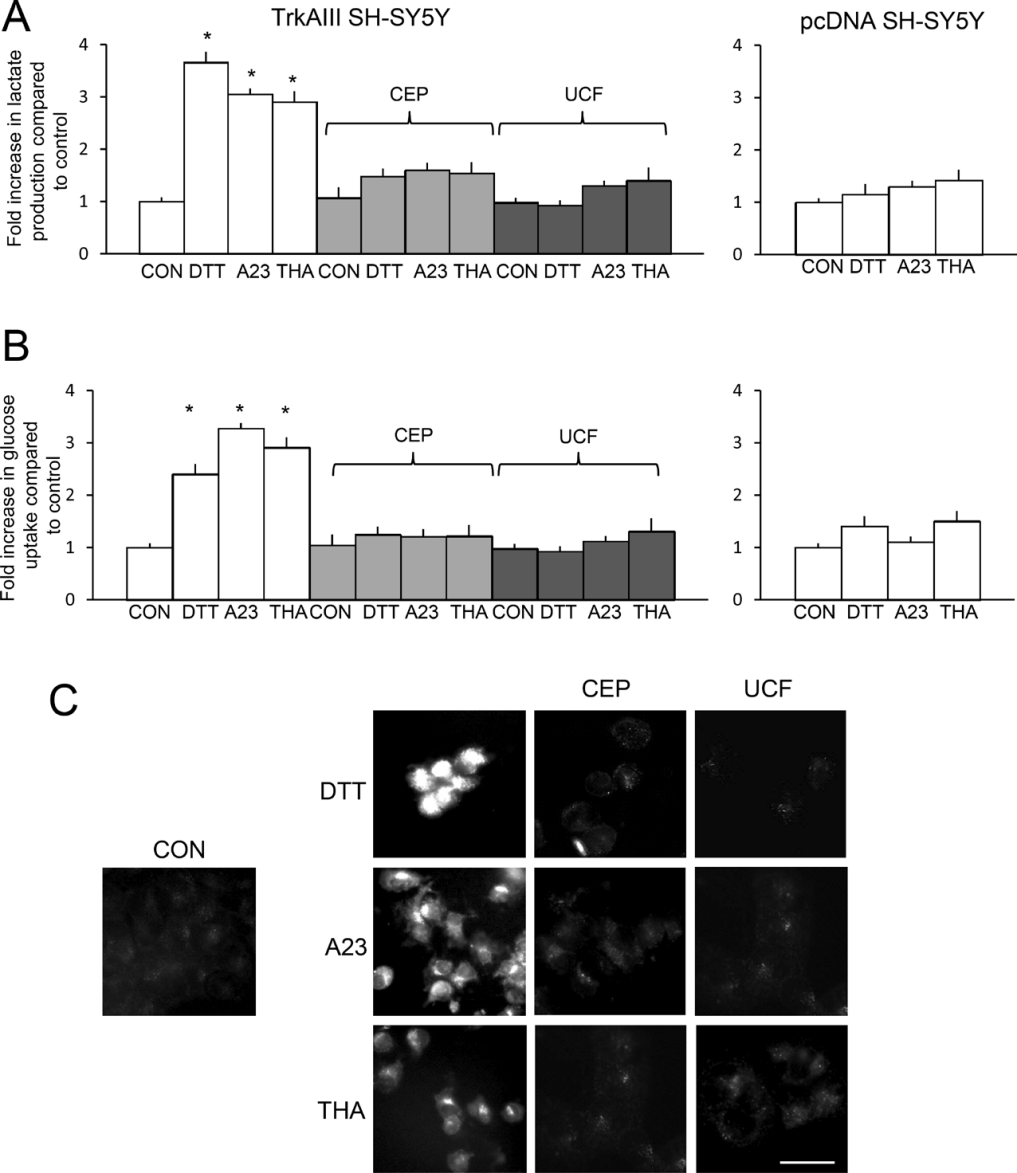
DTT, A23187 and thapsigargin promote aerobic glycolysis in TrkAIII SH-SY5Y cells (**A**) Histograms demonstrating a significant increase (^*^) in lactate production by TrkAIII SH-SY5Y cells, following treatment with DTT (5 mM), A23187 (A23, 10 µM) and thapsigargin (THA, 10 ng/ml) in the absence of inhibitors (white) and no increase in lactate production cells pre-incubated with CEP-701 (CEP, 100 nM) (light grey) or in cells pre-incubated with Ucf-101 (UCF, 10 µM, dark grey) (left histogram) plus no significant increase in pcDNA SH-SY5Y lactate production following treatment with DTT (5 mM), A23187 (A23, 10 µM) and thapsigargin (THA, 10 ng/ml) (right histogram). Results are displayed as mean (± s.e.) fold difference compared to untreated controls (arbitrary value of 1) in 3 independent experiments, each performed in duplicate. (**B**) Histograms demonstrating a significant increase (^*^) in glucose uptake by TrkAIII SH-SY5Y, following treatment with DTT (5 mM), A23187 (A23, 10 µM) and thapsigargin (THA, 10 ng/ml) in the absence of inhibitors (white) but not in cells pre-incubated with CEP-701 (CEP, 100 nM) (light grey) or with Ucf-101 (UCF, 10 µM, dark grey) (left histogram), plus no significant increase in pcDNA SH-SY5Y glucose uptake following treatment with DTT (5 mM), A23187 (A23, 10 µM) and thapsigargin (THA, 10 ng/ml) (right histogram). Results are displayed as the mean (± s.e.) fold increase in glucose uptake compared to untreated controls (arbitrary value of 1), in three independent experiments, each performed in duplicate (^*^ = statistical significance). (**C**) Fluorescent micrographs demonstrating increased 2-NBDG uptake by TrkAIII SH-SY5Y cells, following 6 hour treatment with DTT (5 mM), A23187 (A23, 10 µM) and Thapsigargin (THA, 10 ng/ml) compared to untreated TrkAIII SH-SY5Y cells (CON), plus CEP-701 (CEP, 100 nM, 3 hour pre-incubation) and Ucf-101 (UCF, 10 µM, 3 hour pre-incubation) inhibition of 2-NBDG uptake in DTT, A23187 and thapsigargin-treated TrkAIII SH-SY5Y cells, in a 1 hour uptake assay (bar = 50 μm).

DTT (5mM for 6 hours), A23187 (10 µM for 6 hours) and thapsigargin (10 ng/ml for 6 hours) significantly increased TrkAIII SH-SY5Y lactate production by a mean (± s.e.) of 3.66 ± 0.21 fold for DTT (*P* < 0.0001, df = 10), 3.05 ± 0.11 fold for A23187 (*P* < 0.0001, df = 10) and 2.91 ± 0.23 fold for thapsigargin (*P* < 0.0001, df = 10) (Figure 9A). Pre-incubation with CEP-701 (100 nM, for 3 hours) or Ucf-101 (10 µM, for 3 hours) completely prevented DTT, A23187 and thapsigargin stimulation of TrkAIII SH-SY5Y lactate production (Figure 9A, left histogram). DTT, A23187 and thapsigargin did not significantly increase pcDNA SH-SY5Y lactate production (Figure 9A, right histogram).

In 1-hour glucose (2-NBDG) uptake assays, DTT (5mM for 6 hours), A23187 (10µM for 6 hours) and thapsigargin (10ng/ml for 6 hours) all significantly increased TrkAIII SH-SY5Y glucose uptake by a mean (± s.e.) of 2.4 ± 0.21 fold for DTT (*P* = 0.002, df = 10), 3.27 ± 0.11 fold for A23187 (*P* < 0.001, df = 10) 2.91 ± 0.24 fold for thapsigargin (*P* < 0.0001, df = 10) (Figure 9B). Pre-incubation with CEP-701 (100 nM, for 3 hours) or UCF-101 (10 µM, for 3 hours) also completely prevented DTT, A23187 and thapsigargin stimulation of TrkAIII SH-SY5Y glucose uptake (Figure 9B). DTT, A23187 and thapsigargin did not significantly increase glucose uptake by pcDNA SH-SY5Y cells (Figure 9B, right histogram).

Fluorescence microscopy confirmed the increased in 2-NBDG-uptake by TrkAIII SH-SY5Y cells, following treatment with DTT (5 mM), A23187 (10 µM) and thapsigargin (10 ng/ml). This was not detected in DTT-treated TrkAIII SH-SY5Y cells pre-incubated with CEP-701 (100 nM, for 3 hours) or Ucf-101 (10 µM, for 3 hours) (Figure 9C). Together these data implicate Omi/HtrA2 and TrkAIII activity in ER stress promotion of aerobic glycolysis in TrkAIII SH-SY5Y cells.

### TrkAIII enhances SH-SY5Y resistance to ER stress-induced apoptosis

**Figure 10:**
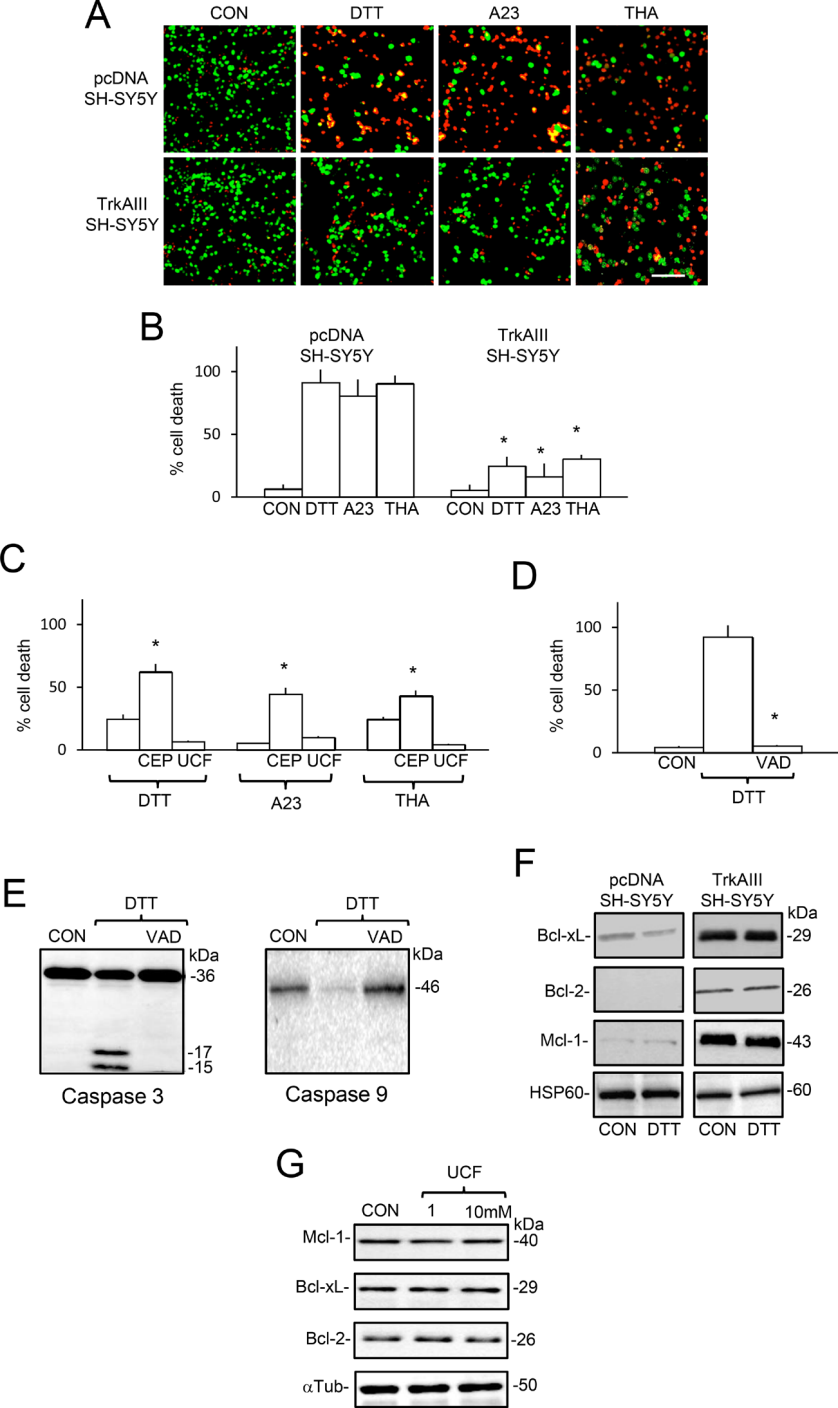
TrkAIII augments SH-SY5Y cell-resistance to DTT, A23187 and thapsigargin-induced death (**A**) Micrographs demonstrating increased death of pcDNA SH-SY5Y cells compared to TrkAIII SH-SY5Y cells (red = dead, green = alive), following treatment with DTT (5 mM for 6 hours), A23187 (A23, 10 µM for 6 hours) and thapsigargin (THA, 10 ng/ml for 6 hours) in 16 hour assays (bar=100 μm). Histograms displaying: (**B**) significant differences (^*^) in TrkAIII SH-SY5Y cell death compared to pcDNA SH-SY5Y cell death, following treatment with DTT (5 mM), A23187 (A23, 10 µM) and thapsigargin (THA, 10 ng/ml); (**C**) significant (^*^) increase in DTT-induced TrkAIII SH-SY5Y death in cells pre-incubated with CEP-701 (100 nM, CEP) but not in cells pre-incubated with Ucf-101 (UCF, 10 µM) and (**D**) significant (^*^) inhibition of DTT-induced pcDNA SH-SY5Y death by z-VAD-fmk (VAD, 10µM). Results are expressed as mean (± s.e.) percent cell death in three independent experiments, each performed in duplicate. (**E**) Western blots demonstrating cleavage of caspase 3 and caspase 9 in pcDNA SH-SY5Y cells treated with DTT (5mM for 6 hours) but not in untreated (CON) or DTT-treated pcDNA SH-SY5Y cells pre-incubated with z-VAD-fmk (10 µM, VAD) (20 μg/lane). (**F**) Western blots demonstrating elevated levels of Bcl-xL, Bcl-2 and Mcl-1 but not Hsp60 proteins, in mitochondria from untreated and DTT-treated (5 mM for 6 hours) TrkAIII SH-SY5Y cells compared to pcDNA SH-SY5Y cells (20 μg/lane). (**G**) Western blots demonstrating that Ucf-101 (1 and 10 µM for 16 hours) does not reduce Mcl-1, Bcl-2 or Bcl-xL, relative to α-tubulin expression in TrkAIII SH-SY5Y cells.

In 16-hour cell death assays, treatment of pcDNA SH-SY5Y cells with DTT (5 mM), A23187 (10 µM) and Thapsigargin (10 ng/ml) induced a mean (± s.e.) of 91.4 ± 10.2% death for DTT (*p* < 0.0001, df = 10), 80.5 ± 13.2% death for A23187 (*P* = 0.0003, df = 10) and 90.4 ± 6.5% death for thapsigargin (*P* < 0.0001, df = 10). In contrast, treatment of TrkAIII SH-SY5Y cells with DTT (5 mM), A23187 (10 µM) and thapsigargin (10 ng/ml) induced a mean (± s.e.) of 24.8 ± 7.2% death for DTT, no significant death for A23187 (*p* = 0.71, df = 10) and 12.5 ± 3.4% death for thapsigargin cell (Figure 10A and 10B). Pre-incubation of TrkAIII SH-SY5Y cells with CEP-701 (100 nM) but not Ucf-101 (10 µM) significantly increased DTT, A23187 and thapsigargin-induced death to 62.2 ± 3.6% (*P* = 0.022, df = 8), 44.5 ± 11.3% (*P* = 0.038, df = 10) and 43.1±7.6% (*P* = 0.03, df = 10), respectively (Figure 10C).

DTT-induced pcDNA SH-SY5Y death was abrogated by z-VAD-fmk (10µM) (Figure 10D) and associated with caspase 9 and caspase-3 cleavage, which was not detected in untreated pcDNA SH-SY5Y cells or DTT-treated cells pre-incubated with z-VAD-fmk (Figure 10E), confirming caspase-dependent apoptosis. Western blots detected higher levels of Bcl-xL, Bcl-2 and Mcl-1 proteins, relative to HSP60, in mitochondria purified from TrkAIII SH-SY5Y compared to pcDNA SH-SY5Y cells (Figure 10F). We have previously shown that TrkAIII inhibitors reduce Bcl-2 and Bcl-xL expression in TrkAIII SH-SY5Y cells [54]. In contrast, Ucf-101 (1 and 10 uM for 16 hours) did not significantly reduce Mcl-1, Bcl-xL or Bcl-2 protein expression in TrkAIII SH-SY5Y (Figure 10G). Together, these data implicate extra-mitochondrial TrkAIII activity rather than mitochondrial Omi/HtrA2 or TrkAIII activity in augmenting Bcl2 and Bcl-xL expression and survival in TrkAIII SH-SY5Y cells.

## DISCUSSION

In this study, we report a novel stress-activated, metabolism-regulating role for the TrkAIII oncoprotein in SH-SY5Y NB cells. We show that TrkAIII not only enhances survival under ER stress conditions but signals ER stress to the mitochondria, resulting in glycolytic metabolic adaptation. ER stress induced by DTT, A23187 and thapsigargin promoted TrkAIII targeting to the mitochondria and internalisation into IMMs, resulting in Omi/HtrA2-dependent cleavage-activation of TrkAIII to active CT-fragments in predominantly tyrosine kinase domain mitochondrial matrix orientation. We provide evidence that this process depends upon a stress-induced increase in ROS production underpinned by changes in Ca^2+^ movement and that stress-induced cleavage-activation of mitochondrial TrkAIII results in the tyrosine phosphorylation of mitochondrial PDHK1, leading to a glycolytic metabolic adaptation. Finally, we show that the TrkAIII inhibitor CEP-701 and the Omi/HtrA2 inhibitor Ucf-101 prevent this mechanism.

TrkAIII targeting to the mitochondria could not be explained simply on the basis of a NT mitochondrial translocation sequence, which was calculated online with a low probability (0.2) of mitochondrial translocation (http://mitf.cbrc.jp/MitoFates/cgi-bin/top.cgi), implicating an atypical mechanism. Furthermore, mitochondrial TrkAIII was neither poly-mono-ubiquinated nor poly-ubiquitinated (data not shown), suggesting an alternative localisation mechanism to that reported for ubiquitinated p53 [55]. The TrkAIII inhibitor CEP-701 did not reduce mitochondrial TrkAIII levels, indicating a TrkAIII activity-independent mechanism and the Hsp90 inhibitor Geldanamycin-A also failed to reduce mitochondrial TrkAIII levels, indicating that Hsp90, which binds TrkAIII [3] and regulates mitochondrial protein importation through TOM-70 [56], was also not involved. Potential involvement of the ER chaperone Grp78/Bip, which also binds TrkAIII [3] and exhibits ER stress-induced mitochondrial translocation [57], is under investigation. Alternatively, TrkAIII may be recognised as a damaged or stress-protein and targeted to the mitochondria for degradation [58]. This possibility is supported by the detection of 30-35kDa CT TrkAIII degradation fragments in mitochondrial matrices under both non-stress and ER-stress conditions.

TrkAIII was not only detected in purified mitochondria but also in ER membranes and MAMs. TrkAIII in purified MAMs, positive for TOM20 and calnexin but not α-tubulin as previously reported [44, 45], was not enriched compared to ER membranes and mitochondria, confirming its association with all three compartments [this study, 3, 11]. The presence of TrkAIII in MAMs suggests that ER-associated TrkAIII may gain access to mitochondria via these sites.

Proteinase K digestion of mitochondrial TrkAIII to an 18kDa CT fragment, resistant to further degradation in the presence IMM permeabilizing digitonin concentrations, suggests that this hydrophobic domain of TrkAIII [59] may be anchored and inserted within the OMM bilayer, protecting it from further degradation.

In contrast to non-mitochondrial TrkAIII, mitochondrial TrkAIII was not constitutively Y490 or Y674/5 tyrosine phosphorylated under non-stress conditions. This indicates that mitochondria have a higher threshold for spontaneous TrkAIII activation than either ERGIC/COP1 membranes or the centrosome [1, 11, 12]. A potential role for mitochondrial PTPases in maintaining this high threshold was supported by detection of the TrkA-de-phosphorylating PTPases SHP-2 and PTP1B [60, 61] in purified mitochondria and by rapid activation of mitochondrial TrkAIII by the PTPase inhibitors sodium orthovanadate and H_2_O_2_ [62, 63]. NGF, on the other hand, could not activate mitochondrial TrkAIII.

Treatment of TrkAIII SH-SY5Y cells with the ER stress inducers DTT, A23187 and thapsigargin activated the ERSR, increased TrkAIII targeting to mitochondria and promoted the mitochondrial TrkAIII internalisation into IMMs. Mitochondrial TrkAIII targeting may involve Grp78/bip, which binds TrkAIII [3] and exhibits ER stress-induced translocation from the ER to mitochondria [57]. Stress-induced mitochondrial TrkAIII internalisation, on the other hand, may involve Omi/HtrA2, since the Omi/HtrA2 inhibitor Ucf-101 [46] reduced mitochondrial TrkAIII levels under conditions of ER stress. We are further investigating these possibilities.

Stress-induced internalisation of mitochondrial TrkAIII into IMMs was associated with TrkAIII cleavage to 45-48kDa active Y490 and Y674/5 phosphorylated CT fragments in tyrosine kinase domain mitochondrial matrix orientation, suggesting that TrkAIII signals ER stress to the mitochondrial matrix. Focussing upon the effect of DTT, mitochondrial TrkAIII cleavage-activation was prevented by both Ucf-101 and siRNA Omi/HtrA2 knockdown, implicating Omi/HtrA2. At present, it remains unclear whether Omi/HtrA2 degrades TrkAIII directly or indirectly via activation of other enzymes. Furthermore, A23187 induced mitochondrial TrkAIII cleavage to a 45kDa active CT fragment rather than the 48kDa active CT fragments induced by DTT and thapsigargin, suggesting involvement of additional and/or alternative proteases. A23187 also reduced the size of full-length mitochondrial TrkAIII to 95kDa, detected by an antibody against the TrkA CT-terminus, suggesting that it may promote additional cleavage at the TrkAIII CT terminus. Ucf-101, however, abrogated TrkAIII activation by all three agents, supporting a common role for Omi/HtrA2. In contrast to Omi/HtrA2, we found no evidence for the involvement of caspases, MMPs or ADAMS in DTT-induced cleavage-dependent mitochondrial TrkAIII activation. Together, these data suggest that Omi/HtrA2 plays a central role in stress-induced mitochondrial TrkAIII cleavage-activation, providing a novel addition to ADAMs/secretase-dependent cleavage-activation of fully-spliced cell surface TrkA [64]. Furthermore, Ucf-101 not only abrogated DTT-induced TrkAIII cleavage but also phosphorylation, implying a cleavage-dependent activation mechanism, presumably facilitated by the elimination of remaining spontaneous activation-prevention domains from TrkAIII [9]. This unveils a novel role for mitochondrial Omi/HtrA2 in stress-protection [31, 32]. Whether this mechanism represents a ubiquitous response to ER stress, remains to be fully established. TrkAIII cleavage-activation was induced to varying degrees by other ER stress inducers such as brefeldin A, 2-deoxyglucose and DDT-ox but was not induced by the N-glycosylation blocker tunicamycin (data not shown), suggesting that this mechanism is not entirely ubiquitous. However in contrast to the other agents, tunicamycin promoted TrkAIII de-glycosylation to a 70kDa core protein that remained trapped within the ER (data not shown), which may explain this observation. Furthermore, although tunicamycin promotes Omi/HtrA2 expression [65], its effects upon Omi/HtrA2 activity have not yet been reported.

ROS involvement in cleavage-dependent mitochondrial TrkAIII activation was suggested by increased ROS production induced by DTT, A23187 and thapsigargin, and supported using the anti-oxidant Resveratrol [48], which prevented DTT, A23187 and thapsigargin stimulation of ROS production and abrogated stress-induced mitochondrial TrkAIII cleavage-activation. The observation that H_2_O_2_ activated mitochondrial TrkAIII, suggests that the increase in ROS production induced by ER stress facilitates mitochondrial TrkAIII cleavage-activation by inactivating mitochondrial PTPases. Furthermore, increased mitochondrial ROS production has been reported to activate Mpv17l, an IMM-associated Omi/HtrA2 activator [32].

A Ca^2+^ ionophore (A23187) [49], a SERCA Ca^2+^ pump inhibitor (thapsigargin) [50] and a ryanodine receptor Ca^2+^ channel agonist (DTT) [51] all induced mitochondrial TrkAIII cleavage-activation, suggesting a central role for Ca^2+^ in this process. The ryanodine receptor Ca^2+^ channel inhibitor dantrolene [52] abrogated DTT-induced cleavage and phosphorylation of mitochondrial TrkAIII, indicating that the DTT-effect was ryanodine receptor Ca^2+^ channel-dependent. Unfortunately, we were unable to sufficiently knockdown ryanodine receptor expression to confirm this. In contrast, dantrolene did not inhibit either A23187 or thapsigargin-induced mitochondrial TrkAIII cleavage-activation, consistent with the ryanodine receptor-independent Ca^2+^ mobilising mechanisms of these agents [49, 50]. The IP3R Ca^2+^ channel inhibitor 2APB [53] also failed to inhibit DTT, A23187 or thapsigargin-induced mitochondrial TrkAIII cleavage-activation, confirming an IP3R-independent mechanism for all three agents. Therefore: ER release of Ca^2+^ via ryanodine receptors; inhibition of ER Ca^2+^ replenishment and receptor/channel-independent alterations in Ca^2+^ compartmentalisation, are independently capable of inducing mitochondrial TrkAIII cleavage-activation. Furthermore, increased mitochondrial Ca^2+^ levels have been shown to activate Omi/HtrA2 [66] and augment ROS production [21, 23, 26]. This suggests that Ca^2+^/ROS interplay provides conditions necessary to overcome the mitochondrial TrkAIII activation threshold by activating Omi/HtrA2, cleaving TrkAIII and inhibiting PTPase activity. We are further investigating the molecular pathways involved.

Stress-induced mitochondrial TrkAIII cleavage-activation resulted in the tyrosine phosphorylation of mitochondrial proteins, including PDHK1. Stress-activated mitochondrial TrkAIII tyrosine phosphorylated exogenous recombinant PDHK1 *in vitro* and CEP-701 prevented stress-induced PDHK1 tyrosine phosphorylation and the phosphorylation of recombinant PDHK1 by TrkAIII *in vitro,* characterising PDHK1 as a novel potential TrkAIII substrate. PDHK1 tyrosine phosphorylation has been reported to inhibit the pyruvate dehydrogenase complex (PDC), promote lactate production and induce aerobic glycolysis [67, 68]. Consistent with this, DTT, A23187 and thapsigargin increased lactate production and glucose uptake in TrkAIII SH-SY5Y, indicating a metabolic shift to aerobic glycolysis. This was not observed in control pcDNA SH-SY5Y cells and was prevented by CEP-701 and Ucf-101, implicating both TrkAIII and Omi/HtrA2 activity.

ER stress induced glycolytic adaptation in TrkAIII SH-SY5Y cells was also associated with enhanced resistance to DTT, A23187 and thapsigargin-induced death. CEP-701 increased the sensitivity of TrkAIII SH-SY5Y cells ER stress-induced death, implicating TrkAIII activity in enhancing survival. This adds to previous reports that TrkAIII increases resistance of NB cells to a variety of toxic agents [1, 3, 7]. The induction of caspase-dependent apoptosis in DTT-treated pcDNA SH-SY5Y cells was prevented by z-VAD-fmk and associated with caspase-9 and caspase 3 cleavage, implicating the intrinsic apoptosis pathway and suggesting that TrkAIII must block this pathway. In support of this, TrkAIII SH-SY5Y cells exhibit elevated expression of the intrinsic apoptosis inhibitors Bcl-2, Bcl-xL and Mcl-1 and TrkAIII inhibition reduced Bcl2 and Bcl-xL expression [54]. Here, we report that TrkAIII SH-SY5Y mitochondria contained higher levels of Bcl-2, Bcl-xL and Mcl-1 proteins, providing an explanation for the increased resistance to apoptosis mediated via the intrinsic pathway and increased sensitivity to ER stress-induced death in the presence of CEP-701. In contrast, Ucf-101 did not increase TrkAIII SH-SY5Y sensitivity to ER stress-induced death nor reduce Mcl-1, Bcl-2 or Bcl-xL expression, suggesting that extra-mitochondrial rather than mitochondrial TrkAIII activity is responsible for enhancing Bcl-2 and Bcl-xL expression and survival of TrkAIII SH-SY5Y cells, under conditions of ER stress.

In conclusion, we have identified a novel role for the TrkAIII oncoprotein in signaling ER stress to the mitochondria in NB cells that results in glycolytic metabolic adaptation and associates with enhanced survival. We propose that ER stress promotes TrkAIII targeting to the mitochondria and its internalisation into IMMs. This results in Omi/HtrA2-dependent TrkAIII cleavage-activation that also depends upon Ca^2+^/ROS interplay, which provides the conditions necessary to overcome the mitochondrial TrkAIII activation threshold by activating mitochondrial Omi/HtrA2 and inhibiting mitochondrial PTPases. The activation of this mechanism results in tyrosine phosphorylation of mitochondrial PDHK1, leading to a glycolytic metabolic adaptation. We propose that this represents a novel self-perpetuating, drug-reversible, mechanism through which tumour microenvironmental stress may maintain the metastasis promoting “Warburg effect” in TrkAIII expressing NBs.

## MATERIALS AND METHODS

### Reagents and cell lines

Rabbit polyclonal anti-human α-tubulin (H-300), Mcl-1 (S-19), XIAP (H-202), Cytochrome c (H-140), TrkA (C-14), Omi/HtrA2 (H-60) and mouse monoclonal anti-phosphotyrosine (PY99) antibodies were purchased from Santa Cruz (Santa Cruz, CA). Mouse monoclonal anti-human calnexin (ab2798) and Bcl2 (ab692) antibodies and rabbit polyclonal anti- human Bcl-xL (ab2568) antibody were purchased from AbCam (Amersham, UK). Mouse monoclonal anti-human PTP1B (Ab-2) and rabbit polyclonal anti-human SHP-2 (ST1083) antibodies were purchased from Calbiochem (Merck-Millipore, Milan, IT). Rabbit polyclonal anti-human Y234 phosphorylated PDHK1 antibody (AB11597) was from AbSci (College Park, MD). Rabbit polyclonal anti-human PDHK1 (C47-H1), Y490 phosphorylated TrkA (Phospho TrkA Tyr Y490), Y674/675 phosphorylated TrkA (Phospho TrkA Tyr Y674/675) and caspase 3 (9662) were from Cell Signaling Technology (Danvers, MA). Rabbit polyclonal anti-human TrkA (MGR12) was from Alexis Biochemicals (San Diego, CA). Mouse monoclonal anti-human MnSOD (611581) and TOM20 (612278) antibodies were from BD Transduction Laboratories (San Jose, CA). Rabbit polyclonal anti-human HSP60 (HSPD1) antibody was from Sigma-Aldrich (St Louis, MO). Conjugated secondary antibodies were from Jackson Laboratories (Bar Harbor, ME). Thapsigargin, HEPES, sodium orthovanadate, hydrogen peroxide, NGF (NO513), DTT, A23187, dantrolene, 2-APB, proteinase K, digitonin, z-VAD-fmk, Ucf-101, TAPI-2, Percoll, EGTA, acridine orange and ethidium bromide were from Sigma-Aldrich (St Louis, MO). Resveratrol was from ENZO Life Sciences (3V Chimica, Rome, IT). Zeocin was from Thermo Fisher Scientific (Milan, IT). Hybond C-extra nitrocellulose membranes and chemiluminescence ECL kits were from Amersham International (Bedford, UK). Cell-based glycolysis and glucose uptake kits were from Cayman Chemicals (Ann Arbour, MI). TriFect Omi/HtrA2-specific dicer substrate RNAi kit was from Integrated DNA Technologies (Bologna, IT). Focus Sub Cell Mitochondrial isolation kits were from G-Biosciences (St. Louis, MO). MitoTracker Red and MitoSox Red reagents were from Invitrogen (Thermo Fisher Sci., Milan, IT). CEP-701 was from Cephalon Incorporated (West Chester, PA).

TrkAIII SH-SY5Y and pcDNA SH-SY5Y cell lines were obtained from SH-SY5Y neuroblastoma cells (ATCC CRL2266), have been described previously [1] and were routinely cultured in DMEM supplemented with 10% foetal calf serum, glutamine and antibiotics (penicillin, streptomycin for all cultures plus Zeocin).

### Purification of mitochondria, mitoplasts and MAMs

Mitochondria were purified using a Focus Sub cell Mitochondrial isolation kit, as described by the manufacturer (G-Biosciences, St. Louis, MO). Briefly, cells were harvested in ice cold PBS by scraping, centrifuged at 1,200 × g for 5 minutes at 4°C, 500 μl of ice cold Buffer I, containing 1× protease inhibitor cocktail (Sigma) was added to the pellet and cells were disrupted by 10 passages through a 20 gauge needle. 250 μl of Buffer II was then added to the homogenate and samples were then centrifuged at 1,200 × g for 5 minutes, at 4°C. Supernatants were transferred to fresh tubes, centrifuged at 15,000 x g for 10 minutes at 4°C and the resulting mitochondrial-rich pellet was washed with 500 μl of Buffer II, centrifuged at 15,000 × g for 10 minutes at 4°C and re-suspended in mitochondrial storage buffer (250mM mannitol, 5-mM HEPES (pH 7.4). Crude mitochondrial preparations in 250mM mannitol, 5mM HEPES (pH 7.4) and 0.5mM EGTA were then separated by Percoll density gradient centrifugation, as previously described [69]. Briefly, crude mitochondrial preparations were layered onto a 30% Percoll gradient in the same buffer and ultra centrifuged at 90,000 × g for 40 minutes at 4°C. Ultrapure mitochondria were collected from a band located 2/3rds from the top, transferred to a fresh tube, diluted 1:10 in 250 mM mannitol, 5 mM HEPES (pH 7.4) containing 0.5 mM EGTA and re-centrifuged at 15,000 × g for 10 minutes, at 4°C. Mitochondrial pellets were then re-suspended in the desired volume of cold 250 mM mannitol, 5-mM HEPES (pH 7.4) containing 0.5 mM EGTA immediately prior to experimentation.

Mitoplasts were obtained from purified mitochondria as previously described [70]. Briefly, ultra purified mitochondria were incubated with 0.1 μg digitonin/ml of mitochondrial protein with gentle stirring on ice for 15 minutes. Three volumes of 250 mM sucrose in mannitol/HEPES/EGTA pH [7.4] buffer were then added. Mitoplasts were pelleted by centrifugation at 15,000 × g for 10 minutes and re-suspended in SDS PAGE reducing sample buffer.

MAMs were purified as previously described [43]. Briefly, cells (10^9^) were harvested, washed in PBS, pelleted at 500 × g for 5 min, re-suspended in homogenization buffer (0.25 M sucrose and 10 mM HEPES pH 7.4) and dounce-homogenized. Homogenates were centrifuged twice at 600 × g for 5 min to remove cellular debris and nuclei, and supernatants centrifuged at 10,300 × g for 10 min to pellet crude mitochondria. Mitochondria, re-suspended in 250 mM mannitol, 5 mM HEPES (pH7.4), and 0.5 mM EGTA, were layered on top of 8 ml of Percoll medium (225 mM mannitol, 25 mM HEPES (pH 7.4), 1 mM EGTA and 30% Percoll (v/v)) in a 10-ml polycarbonate ultracentrifuge tube and centrifuged for 30 min at 95,000 × g. MAMs, removed as a diffuse white band above mitochondria, were diluted in isolation medium, centrifuged at 6,300 × g for 10 min and the supernatant containing MAMs was centrifuged at 100,000 × g for 1h in a Beckman 70 Ti rotor. The resulting MAM pellet was re-suspended in homogenisation buffer, protein concentrations measured and examined by reducing SDS PAGE Western blotting. Purified MAMs were positive for calnexin and TOM20 but negative for α-tubulin [44, 45], ER fractions were positive for calnexin but negative for TOM20 and α-tubulin, mitochondrial fractions were positive for TOM20 but negative for calnexin and α-tubulin and membrane-free cytosol fractions prepared by 100,000 × g ultracentrifugation, were negative for calnexin and TOM20 but positive for α-tubulin. For proteinase K digestion, purified mitochondria or mitoplasts were incubated with Proteinase K (10 μg/ml) at 0°C for 30 minutes in the presence or absence of digitonin (0.1-0.5 μg/ml), reactions were stopped with 2mM PMSF, digested mitochondria and mitoplasts were pelleted by centrifugation at 15,000 × g for 10 minutes at 4°C, re-suspended in reducing SDS-PAGE sample buffer and analysed by Western blot.

### RNA purification and RT-PCR

RT reactions were performed on total RNAs (1μg), purified using RNA-easy Plus, as described by the manufacturer (Qiagen), using the Moloney Murine Leukemia virus RT kit, as detailed by the manufacturer (LifeTechnologies, Inc, Paisley, UK). RT reactions were subjected to PCR using the following primers: GAPDH: 5′-AGGTCCACCACTGACAGTT-3′ (forward) and 5′-CTGCACCACCAACTGCTT AG-3′ (reverse); CHOP: 5′-ACCAAGGGAGAACCAGGAAACG-3′ (forward) and 5′-TCACCATTCGGTCAAT CAGGC-3′ (reverse); XBP1: 5′-TTACGAGAGAAAACTCATGGC-3′ (forward) and 5′-CGGTCCAAGTTGTCCAGA ATGC-3′ (reverse).

### Labeling mitochondria with MitoTraker-Red and MitoSox-Red

Cells grown on glass chamber slide (Nunc) were treated with reagents at concentrations and for the times indicated in the text and figure legends. Following incubation, cells were washed 5 times with pre-warmed PBS (37°C), particularly important for DTT which inhibits reagent fluorescence, and either MitoTraker-red (for mitochondria localization) or Mitosox red (for ROS detection) solution added to a final concentration of 2.5μM, and incubated for 15 minutes at 37°C. Cells were then washed three times in pre-warmed PBS, mounted in PBS/Glycerol and examined immediately under a Zeiss “Axioplan-2” fluorescence microscope. Representative fields were digitally photographed under identical exposure conditions.

### SiRNA knockdown of Omi/HtrA2

Knockdown of mitochondrial Omi/HtrA2 expression was achieved using a TriFECTa Dicer-Substrate RNAi kit, employing three Omi/HtrA2-specific Dicer-Substrate siRNA duplexes, as described by the manufacturer (Integrated DNA Technologies, Coralville, IA). Briefly, cells were subjected to 48-hour transient transfection with 50nM of negative control siRNA duplex (provided with the kit) or 50nM of a mix of Omi/Htr2a specific siRNA duplexes, using INTERFERin *in vitro* siRNA transfection reagent, as described by the manufacturer (Polyplus Transfection Inc., New York, NY). Sham transfected controls received transfection reagent alone. SiRNA knockdown of Omi/HtrA2 protein expression was confirmed in purified mitochondrial extracts by Western blot comparison to HSP60 levels. Transfection efficiency was confirmed using a HPRT-S1 DS positive control and validated using a negative control duplex (NC1) not present in the human genome, as described by the manufacturer (Integrated DNA Technologies; www.IDTDNA.com). The Omi-specific siRNA duplexes were as follows:

5′-ACAUCGCAACGCUGAGGA-3′ and 5′-GUCU GAAUCCUCAGCGUU-3′ 5′-AGACUGCUAAGCGG CGA-3′ and 5′-CAUACGUGUCGCCGCUUA-3′5′-AGUCAGUACAACUUCAUC-3′ and 5′-CAUCUGCG AUGAAGUUGU-3′

### Inhibitor studies

In inhibitor studies, cells were pre-incubated with aprotinin, z-VAD-fmk, TAPI-2, EDTA, CEP-701 and Ucf-101 in complete medium for 3 hours prior to the addition of DTT, A23187 or thapsigargin and inhibitors were also present for the duration of incubation with these agents. For Resveratrol studies, cells were pre-incubated for 12 hours with Resveratrol prior to the addition of stress-inducing agents and for the duration of subsequent incubation.

### Immunoprecipitation and Western blotting

Cells or purified mitochondria were extracted in lysis buffer (PBS containing 0.5% sodium deoxycholate, 1% NP40, 0.1% SDS, 1mM sodium orthovanadate, 1mM PMSF, 1 μg/ml of pepstatin A and Aprotinin) and protein concentrations calculated by Bradford protein concentration assay (Sigma-Aldrich). Samples for SDS-PAGE were mixed with reducing SDS-PAGE sample buffer and subjected to reducing SDS-PAGE/Western blotting. Briefly, proteins separated by reducing SDS-PAGE, were trans-blotted onto Hybond C+ nitrocellulose membranes by electrophoresis (Amersham Int. UK) and the membranes subsequently air-dried. Non-specific protein binding-site on membranes were blocked by incubation for 2 hours in 5% non-fat milk in TBS-T prior to incubation with primary antibodies, at recommended dilutions, for 2–16 hours at 4°C. Membranes were then washed in TBS-T, incubated with secondary HRP-conjugated antibodies (Jackson ImmunoResearch Laboratories, West Grove, PA) diluted in blocking solution and immunoreactive species detected by chemiluminescence reaction, as directed by the manufacturer (Amersham Int). For immunoprecipitation, extracts (200–500 μg) pre-cleared with IgG and protein sepharose A were incubated with primary antibody at a concentration of 0.1–1.0 μg/500 mg total protein for 2–16 hours at 4°C. Following incubation, 20 μl of Protein A Sepharose (Fast flow, Sigma-Aldrich) in lysis buffer, was added and incubated for 30 minutes at 4°C, with rotation. Protein sepharose/IgG conjugates were then collected by centrifugation (10,000 × g for 5 minutes), washed 3 times in lysis buffer, re-suspended in SDS-PAGE sample buffer and subjected to reducing SDS-PAGE/Western blotting.

### Cell death assay

Cell death assays were performed as previously described [7]. Briefly, cell cultures were washed once in Ca^2+^ free PBS, detached with ice cold PBS containing 1mM EDTA, transferred to sterile 15 mls tubes, centrifuged for 5 minutes at 1,000× g at 4°C, washed again in ice cold PBS and re-pelleted by centrifugation at 1,000 × g for 5 minutes at 4°C. Cell pellets were re-suspended in 25 μl of PBS containing 2 μl of acridine orange/ethidium bromide solution (100 μg/ml acradine orange and 100 μg/ml ethidium bromide in PBS) plated onto glass slides and examined immediately under a Zeiss “Axioplan-2” fluorescence microscope. Representative fields were digitally photographed under identical exposure conditions and the number of dead cells (orange/red nuclei) and live cells (green nuclei) counted.

### Indirect immunofluorescence

Cells grown on Nunc glass chamber slides (Sigma-Aldrich) were washed in PBS, fixed and permeabilized in 100% ice cold methanol (–20°C), washed in PBS then processed for indirect immunofluorescence (IF). Fixed, permeabilized cells were incubated for 1 h in blocking solution (1% bovine serum albumin in PBS-0.03% TX100) and then incubated for 2 hours with primary antibody in blocking solution, at room temperature. Slides were then washed three times in PBS-0.03% TX100, incubated with secondary fluorochrome-conjugated antibody diluted in blocking solution for 1 h at room temperature, washed in PBS-0.03% TX100 and mounted using VectorMount™, containing DAPI nuclear counterstain. Images were obtained using a Zeiss Axioplan 2 fluorescence microscope with digital camera and Leica M500 Image Manager software.

### Glycolysis assay

Glycolysis was measured by L-Lactate production using a cell-based glycolysis assay kit, as described by the manufacturer (Cayman chemical). Briefly, 1 × 10^5^ cells per well were plated onto 96 well plates for glycolysis assays and parallel cell counting, and grown for 12 hours. At 12 hours, cell counts were performed on parallel cultures and duplicate experimental cultures (and parallel cell count cultures) incubated with reagents (medium alone, DTT, A23187 or thapsigargin with or without pre-incubation with inhibitors at the concentrations and times indicated in the text, in a volume of 100 ml complete culture medium, for 6 hours at 37°C. At 6 hours, cell counts were performed on parallel cultures, and in experimental cultures supernatants were removed, cultures were washed 5 times in 150 ml of pre-warmed PBS, 100 ml of fresh medium (containing 1%FCS) added to each well and then incubated for 24 hours at 37°C. At 24 hours, cells were counted and supernatants were removed, centrifuges at 14,000 rpm to remove cell debris and L-Lactate levels were measured in duplicate 10 ml aliquots of culture supernatant and compared to an L-lactate standard curve, as directed by the manufacturer (Cayman Chemicals). Exponential growth rates were calculated from cell counts using the online exponential growth rate calculator at http://www.rapidtables.com/calc/math/ exponential-growth-calculator.htm. Growth rates (units per hour) were used to calculate lactate production rates using the general equation: Production Rate = m(Cf Ci) / (Df  Di), where m = growth rate, Cf = final lactate concentration, Ci = the initial Lactate concentration, Df = final cell density and Di = initial cell density [71]. Lactate production rates were calculated as M lactate/ 100 cells per hour and data presented as fold difference (± s.e.) compared to untreated controls.

### Glucose uptake assays

Glucose uptake was measured using a cell-based glucose assay kit, as described by the manufacturer (Cayman chemical). Briefly, cells at a concentration of 5 × 10^4^ cells/well were seeded onto 96 well plates (for experiments and parallel cell counts), grown overnight then treated for 6 hours with DTT, A23187 or thapsigargin with or without 2 hour pre-incubation with CEP-701 or Ucf-101, in a volume of 100 μl glucose-free culture medium. 2-NBDG (150 μg/ml) was then added in glucose-free medium and cultures incubated for a further 1 hour. At 1 hour, cultures were centrifuged at 400 × g for 10 minutes at RT, supernatants aspirated, 200ml of cell-based assay buffer added, plates re-centrifuged at 400 × g for 5 minutes at RT, supernatants aspirated, 100 μl of cell-based assay buffer added to each well and plates read promptly in a fluorescence plate reader (excitation/emission = 485/535 nm). Serial dilutions of a 1 mg/ml stock solution of 2-NBDG in assay buffer were used as a reference standard. Fluorescent measurements were adjusted for cell numbers, counted using the phase contrast micrographs prepared immediately prior to fluorescence analysis and are presented as fold difference (± s.e.) to untreated controls. 2-NBDG up-take was also examined by direct fluorescence microscopy in cells cultured on Nunc chamber slides, following identical treatment to that used in glucose uptake assays. At the termination of treatment, cells were washed in pre-warmed PBS, mounted in PBS/Glycerol, visualised under a Zeiss Axioplan 2 fluorescence microscope with digital camera and Leica M500 Image Manager software and light micrographs taken.

### Densitometry, statistical analysis and software

Data were analysed statistically using the Student’s *t*-test, using the online *t*-test calculator at https://www.graphpad.com/quickcalcs/ttest1.cfm, with statistical significance associated with probabilities of ≤ 0.05. Mitochondrial translocation probability was calculated using the online protein N-terminal mitochondrial translocation probability calculator at http://mitf.cbrc.jp/MitoFates/cgi-bin/top.cgi. Exponential growth rates were calculated from cell counts, using the online calculator at http://www.rapidtables.com/calc/math/ exponential-growth-calculator.htm. Densitometric and quantification of co-localisation analyses were performed using Image J Fiji software, with colour pixel counter plugin [72].
